# Treatment with Riluzole Restores Normal Control of Soleus and Extensor Digitorum Longus Muscles during Locomotion in Adult Rats after Sciatic Nerve Crush at Birth

**DOI:** 10.1371/journal.pone.0170235

**Published:** 2017-01-17

**Authors:** Wojciech Zmysłowski, Anna M. Cabaj, Urszula Sławińska

**Affiliations:** 1 Department of Engineering of Nervous and Muscular System, Nałęcz Institute of Biocybernetics and Biomedical Engineering, PAS, Warsaw, Poland; 2 Department of Neurophysiology, Nencki Institute of Experimental Biology, PAS, Warsaw, Poland; Szegedi Tudomanyegyetem, HUNGARY

## Abstract

The effects of sciatic nerve crush (SNC) and treatment with Riluzole on muscle activity during unrestrained locomotion were identified in an animal model by analysis of the EMG activity recorded from soleus (Sol) and extensor digitorum longus (EDL) muscles of both hindlimbs; in intact rats (IN) and in groups of rats treated for 14 days with saline (S) or Riluzole (R) after right limb nerve crush at the 1^st^ (1S and 1R) or 2^nd^ (2S and 2R) day after birth. Changes in the locomotor pattern of EMG activity were correlated with the numbers of survived motor units (MUs) identified in investigated muscles. S rats with 2–8 and 10–28 MUs that survived in Sol and EDL muscles respectively showed increases in the duration and duty factor of muscle EMG activity and a loss of correlation between the duty factors of muscle activity, and abnormal flexor-extensor co-activation 3 months after SNC. R rats with 5, 6 (Sol) and 15–29 MUs (EDL) developed almost normal EMG activity of both Sol and control EDL muscles, whereas EDL muscles with SNC showed a lack of recovery. R rats with 8 (Sol) and 23–33 (EDL) MUs developed almost normal EMG activities of all four muscles. A subgroup of S rats with a lack of recovery and R rats with almost complete recovery that had similar number of MUs (8 and 24–28 vs 8 and 23–26), showed that the number of MUs was not the only determinant of treatment effectiveness. The results demonstrated that rats with SNC failed to develop normal muscle activity due to malfunction of neuronal circuits attenuating EDL muscle activity during the stance phase, whereas treatment with Riluzole enabled almost normal EMG activity of Sol and EDL muscles during locomotor movement.

## Introduction

Generally, injuries to peripheral nerves lead to impairment of voluntary movement and gait abnormalities followed by muscle atrophy. Without pharmacological therapy to aid functional recovery, patients rely solely on surgical treatment and rehabilitation. Unfortunately, functional outcomes in human patients remain poor. Thus, a great amount of research carried out on animal models has focused on the effects of nerve injury on the structure and function of neuronal circuits involved in muscle control as well as on the search for pharmacological therapies that may improve functional outcomes in patients. Studies on the effect of sciatic nerve crush (SNC) are of special interest, because SNC is an excellent model of the type of nerve injury that occurs frequently during delivery disabling infants for life.

Incomplete recovery of motor function in animal models can be attributed to the loss of motor and sensory neuronal axons and receptors inducing a loss of connectivity in neuronal circuits involved in motor control [1, 2]. Depending on the age of the animal at the time of SNC, the number of surviving motoneurons ranges from approximately 15% in newborns to almost 100% in adults [3, 4, 5], with a loss of sensory neurons in newborn rats of 40–60% [6, 7]. Less is known about the impact of peripheral nerve injury on proprioceptive feedback circuits. The function of these circuits may be impaired due to the loss of neurons, proprioceptors and degeneration of axons as well as a failure of regenerating afferents to restore connections and/or misdirection of regenerating axons depending on the method of injury and the age of the animal [1, 2, 8, 9, 10, 11, 12].

The effects of nerve injury on motor functions and the effectiveness of pharmacological treatment and surgical repair on locomotor behavior has been studied using animal models of SNC. The outcome measures such as locomotor speed, step cycle and stance phase durations, BBB scale, Sciatic Functional Index (SFI), ground reaction forces and joint kinematics have been used. However, the sensitivity of these measures is not sufficient to reveal postoperative changes in the performance of individual muscles [13–19]. The measurements of ground reaction forces [20] show that adult rats do not fully recover normal vertical forces until 2 months after SNC. Studies on rats with SNC at the 3^rd^ day a.b. using the SFI, ladder rung and tapered beam tests [21] revealed deficits in the performance of the affected limb observed over 12-weeks in opposition to previous findings suggesting recovery of normal locomotor movement. Kemp and colleagues [21] showed that the neuroprotective agent P7C3 enhanced motor and sensory neuron survival and produced a partial recovery of animal performance. Furthermore, it has been reported in rats with SNC at P3-P30 [22] that locomotor recovery, assessed with the SFI in P7 rats, was worse than in naive animals, and a decrease in the number of motor and sensory neurons was seen at P7 and P30 as well as a severe impairment of muscle function observed when SNC was inflicted before P30. Overall, there is a lack of systematic experimental data on the effect of SNC on the neuronal circuits involved in locomotor control and recovery of locomotor function indicating a need for better measures of outcome assessment.

The analysis of EMG activity of hindlimb muscles has been used in a limited number of studies on rats with SNC at birth [23, 24], partial denervation [25–27] or transection and surgical repair [28, 29]. However, analysis was limited to the duration of activity in individual muscles or a general pattern of EMG activity. The effect of treatment with Riluzole on the recovery of locomotor EMG activity has not been studied. However, it has been established that treatment with Riluzole (the only drug currently used for treating patients with neurodegenerative disease) protects motoneurons against degeneration following peripheral nerve injury in adult and newborn rats [5, 30]. A neuroprotective action of Riluzole on motoneurons and improvement of locomotor function (BBB locomotor score) after co-treatment with GDNF in adult rats after nerve avulsion and re-implantation, has been also reported, as well as enhancement of neurite outgrowth in cultures of adult and neonatal rat dorsal root ganglion neurons [31, 32]. Cabaj and Sławińska [5] studied the effect of treatment with Riluzole in rats with SNC at birth on the numbers of motor units and contractile characteristics of soleus (Sol) and extensor digitorum longus (EDL) muscles and on runway locomotion assessed with SFI reveling that rats with a larger number of MUs and better muscle contractile properties demonstrate a significant reduction of the SFI deficit observed after Riluzole treatment.

The present study was designed to reveal the effect of SNC at birth in one limb in rats on EMG activity of Sol and EDL muscles during locomotion recorded simultaneously in the muscles of both limbs (SNC and contralateral control) providing new data on the impairment of muscle control during locomotion and the effect of Riluzole treatment. Remarkably, with the same number of motor units rescued in their Sol and EDL muscles treatment enabled development of almost normal control of muscles compared to saline treated rats. A portion of these results has been previously presented in abstract form [33].

## Materials and Methods

### Subjects

Wistar rat pups of both sexes were used in these experiments which were carried out with the approval of the First Ethic Committee for animal Experimentation in Poland, according to European Union and Polish law on Animal Protection. The locomotor EMG pattern of some animal has been recorded and presented in a previous study, in which the effects of SNC and treatment with Riluzole on the number of motor units (MUs), contractile characteristics of muscles and Sciatic Functional Index were studied [5].

### Surgical procedures

Two series of experiments were performed in which SNC was inflicted after birth: 6h (1^st^ day a.b.) and 24h (2^nd^ day a.b.). Surgery was performed in sterile conditions on Wistar rat pups anaesthetized by cooling for 10–15 min at -10°C and then kept during surgery on cold surface. A skin incision was made at the mid-thigh region of the right hindlimb and then the sciatic nerve was exposed and crushed. The nerve was crushed for 5s just above the division of the sciatic nerve into the tibial and common peroneal nerves using a fine pair of watchmaker forceps under a surgical microscope to ensure that the nerve was completely crushed but not torn. The skin incision was then sutured [5].

The experimental animals after the SNC were divided into groups: those treated daily with Riluzole (IP, 16 mg/kg/day) until they were 14 days of age (1R: crush at 1^**st**^ day a.b. and 2R: crush at 2^**nd**^ day a.b.) or sterile saline (sodium chloride 0.9%) in the same fashion (1S: crush at 1^st^ day a.b. and 2S: crush at 2^nd^ day a.b.). The first injection was performed immediately after SNC. We used the dose of 16 mg/kg/day (IP) consistent with the findings of Khoo and colleagues [34] that lower doses of Riluzole improved Sol muscle recovery without rescuing any motoneurones. After injection the animals were warmed under a lamp, observed and returned to their mother.

To investigate locomotor abilities, 3 months after SNC the implantation of electrodes for chronic EMG recordings was performed both in rats with SNC (n = 22) and in intact animals (n = 3). The EMG wire electrodes were implanted under sterile conditions into the Sol and EDL muscles of the left and right hindlimbs under Isoflurane (2 l/min of O_2_ + 2% Isoflurane and s.c. Butomidor 0.05 mg/kg b.w.). EMG recordings started 1–2 days after implantation. The electrodes were made from multistranded, Teflon coated stainless steel wire (0.24 mm in diameter; AS633, Cooner Wire, Chashworth, CA, USA) with a multipin connector [35] secured to the back of the animal. The hook electrodes were led under the skin and inserted into the appropriate muscles. In all experiments the distance between the two tips of electrodes was 1–2 mm.

### Electromyographic recordings during locomotion

Before the recording electrodes were implanted the rats were trained to walk along a horizontal runway consisting of a start platform, a 2 m long, 10 cm wide runway and a goal box. A one day session consisting of 10 trials was sufficient to train animals to walk on the runway. Electromyographic activity was simultaneously recorded from the Sol and EDL muscles of the left (L) and right (R) hindlimbs for a few weeks in rats treated with saline (n = 9) and Riluzole (n = 13) after SNC and in intact animals (n = 3). Signals were amplified, filtered (0.1–1 kHz) and recorded on a tape recorder (RACAL V-STORE). For each animal, one experimental session consisted of 10 passes along the runway, with 10 to 12 steps per pass, which gave a sample of EMG data recorded in more than 50 steps collected during each session for every rat. EMG signals were converted a/d with a sampling frequency of 2 kHz.

### Data analysis

Activity of muscles in locomotor movement is often characterized by interlimb and intralimb coordination, cycle duration and burst duration of EMG muscle activity. The structure of the flexor and extensor EMG activity within an individual cycle and the relationship between these variables is often omitted. Thus, EMG recordings were obtained during locomotion for the Sol and EDL muscles of the left (control) and right (with SNC) hindlimbs in rats treated with saline (n = 9) and rats treated with Riluzole (n = 13), as well as in intact animals (n = 3). EMG activity was analyzed to derive: (1) phase shift and strength of inter- and intralimb coordination; (2) cycle duration; (3) burst duration and relative burst duration (duty factor) of muscle EMG activity as well as the correlation coefficients and the parameters of regressions for the relationships between: (4) the burst duration of muscle EMG activity and the cycle duration, (5) the duty factors established for right and left hindlimb (with nerve crush and control) and between (6) the burst duration of EDL muscle EMG activity and the burst duration of Sol muscle EMG activity.

**Fig 1:**
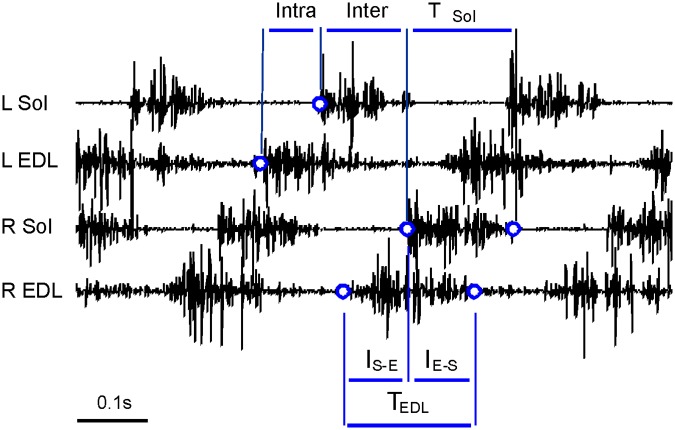
Explanation of time intervals used for data analysis. The vertical lines in the upper part of this figure denote examples of time intervals used to calculate phase shifts of inter- and intralimb coordination in intact rat. The vertical lines at the bottom of figure denote time intervals I_S-E_ and I_E-S_ used in the analysis of relationships between the burst duration of the right EDL muscle and the burst duration of the right Sol muscle EMG activity in intact rat. Abbreviations: L—left, R—right, Sol—soleus, EDL—extensor digitorum longus, Inter-interlimb coordination, Intra-intralimb coordination, T—burst duration of muscle EMG activity.

The duration of the cycle and the burst of muscle EMG activity was calculated for both Sol and EDL muscles of both hind-limbs from the corresponding onset and offset of muscle EMG activity using a semiautomatic program written in our laboratory. The phase shifts of inter- and intralimb coordination were calculated using time intervals between the onsets of EMG bursts of activity in Sol and EDL muscle (Fig 1). The relationship between the burst durations of EDL and Sol muscle EMG activity was analyzed using time interval, I_S-E_, between the onset of activity in the EDL and Sol muscle, and the time interval, I_E-S_, between the onset of activity in the Sol muscle and the offset of activity in the EDL muscle (Fig 1).

#### Interlimb and intralimb coordination

Interlimb coordination between left and right hindlimbs was established for the Sol and EDL muscles with phase shifts between the onset of activity bursts in the left and right muscles of intact rats (Fig 1) as well as in the control muscles and muscles with SNC of saline and Riluzole treated rats. Thus, the phase shifts were calculated as the ratio of time intervals between the onset of muscle activity (R/SNC Sol—L/Co Sol and R/SNC EDL—L/Co EDL) to the cycle duration obtained for the left as well as for the control Sol and EDL muscles. The intralimb coordination was established for both hindlimbs with phase shifts between the onsets of activity bursts in the EDL and Sol muscles of the left and right hindlimbs in intact rats (Fig 1) as well as of the control hindlimbs and hindlimbs with SNC h in saline and Riluzole treated rats. Thus, the phase shifts were calculated as the ratio of time intervals between the onset of muscle activity (L/Co Sol—L/Co EDL and R/SNC Sol—R/SNC EDL) to the cycle duration obtained for the left and right Sol muscles as well as for the control muscles and muscles with SNC. The strength of coordination was determined with the length of vector ***r*** using polar plot analysis [36].

#### The burst duration of muscle EMG activity and the relationship between the burst duration and the cycle duration

The relationship between the burst duration of muscle EMG activity and the cycle duration was assessed quantitatively by regression and correlation analysis to reveal the effects of SNC and treatment with Riluzole on adaptation of burst duration to the cycle duration, which is a prerequisite of normal locomotion.

#### The relationship between the duty factors established for right and left hindlimb (with sciatic nerve crush and control)

The duty factor of burst was included to the analysis in addition to burst duration, because a ratio of time taken by burst, to cycle duration, enabled direct comparison of muscle activity in animals with different cycle durations. The relationship between the duty factors was used to reveal the effects of SNC and treatment with Riluzole, on the adaptation of EMG activity of muscles with SNC to the activity of control muscles using the coefficient of correlation between these variables. A strong correlation between the duty factors of muscle activity bursts is a prerequisite of normal locomotion [37].

#### The relationship between the burst duration of EDL and Sol muscle EMG activity

The effects of SNC and treatment with Riluzole on the burst duration of EDL muscle EMG activity was analyzed assuming that prolongation of the activity observed in saline treated rats was due to the effect of SNC on the relationship between the activity of EDL and Sol muscles. The EDL muscle activity can be described using two time intervals: between the onset of activity in the EDL muscle and the onset of activity in the Sol muscle, I_S-E_, and between the onset of activity in the Sol muscle and the offset of activity in the EDL muscle, I_E-S_ (Fig 1). We analyzed the relationship between these intervals and the burst duration of Sol muscle EMG activity. Regression analysis was used to separate the effect of SNC or treatment with Riluzole on the duration of the interval, from the effect of a change in the Sol muscle activity duration induced by SNC or treatment, with the use of a predicted value. The predicted value is defined as the value which would be observed if a rat with SNC moved with the duration of Sol muscle activity observed in intact rat. According to this definition the predicted value was calculated by extrapolating data obtained in rats treated with saline or Riluzole using the regression line obtained. Thus, the difference between the predicted value of the locomotor indice and the value obtained in intact rat, estimates the proportion of the effect of SNC or treatment due to the action on the relationship characterizing control of interval duration.

### Statistical analysis

Data listed in the text are expressed as the mean ± SD, while the values of SEM are shown numerically in figure legends. Statistical significance of inter- and intralimb coordination was examined by Rayleigh’s test [36]. The normality of data and homogeneity of variances were examined with D’ Agostino-Pearson and Bartlett tests. If the assumptions of normality or homogeneity of variance or both were violated, then the Kruskal-Wallis test was used. The relationships were examined with regression (least square method) and correlation analyses (Pearson) using data from 50 steps obtained in each animal. The correlation coefficients were assessed and compared using Student’s *t* test considerations, Fischer’s *r*-to-*z* transformation and chi-square test for heterogeneity [36]. The difference in the mean, the regression slope and the correlation coefficient was considered statistically significant if *p* < 0.01. Statistical analysis was carried out using STATISTICA (StatSoft^®^) and Excel.

## Results

### The number of motor units

The number of MUs in the Sol and EDL muscles of intact rats and in control muscles of saline and Riluzole treated rats were approximately 28 and 40, respectively. As we described in our previous paper [5], the number of MUs that survived in the Sol muscles of saline treated rats (n = 9) were within the same range of 2–8 (7–28%) after SNC inflicted at 1^st^ and 2^nd^ day a.b. On the other hand in EDL muscles the number of MUs was 10–28 (25–70%) after SNC at 1^st^ day a.b. (n = 4) and 16–28 (40–70%) after SNC at 2^nd^ day a.b. (n = 5). Rats treated with Riluzole after SNC at 1^st^ day (n = 5) and 2^nd^ day (n = 8) showed 5–11 (18–39%) and 5–16 (18–57%) MUs rescued in their Sol muscles, whereas in EDL muscles there were 22–33 (55–82%) and 15–29 (37–72%), respectively (Table 1). The number of MUs surviving in the EDL muscle after SNC was greater than that in Sol muscle consistent with the previous findings [3, 5] that motoneurons to EDL muscle mature earlier than those to the Sol.

**Table 1:** Motor unit numbers in the Sol and EDL muscles in rats treated either with saline or Riluzole after SNC inflicted at 1^st^ and 2^nd^ day after birth (a.b.).

Group	1S (n = 4)	Sol	EDL	2S (n = 5)	Sol	EDL
	NB4	2	25	NA4	2	22
	NB5	4	15	NA5	4	28
	NB2	6	10	NA7	6	16
	NB6	**8**	**28**	NA6	**8**	**24**
				KB6	**8**	**25**
Group	1R (n = 5)	Sol	EDL	2R (n = 8)	Sol	EDL
R G1	RB4	5	29	RA1	5	15
	RB5	6	26	RA4	6	20
(n = 5)				RA6	6	22
R G2	RB6	8	33	RA5	**8**	**26**
(n = 4)	RB7	**8**	**25**	RA11	**8**	**23**
	RB3	11	22	RA9	14	29
				RA8	15	17
				RA10	16	21

The table contains numbers of motor units in the Sol and EDL muscles in rats treated with saline and Riluzole after SNC at 1^st^ and 2^nd^ day a.b. Abbreviations: Sol—soleus, EDL- extensor digitorum longus, 1S and 2S –rats treated with saline after SNC at 1^st^ and 2^nd^ day a.b., 1R and 2R—rats treated with Riluzole after SNC at 1^st^ and 2^nd^ day a.b., RG1—rats treated with Riluzole which developed normal activity of Sol muscles, RG2—rats treated with Riluzole which developed normal activity of Sol and EDL muscles. Data corresponding to subgroups of saline and Riluzole treated rats with similar numbers of motor units in their Sol and EDL muscles are denoted in bold.

### General observations

**Fig 2:**
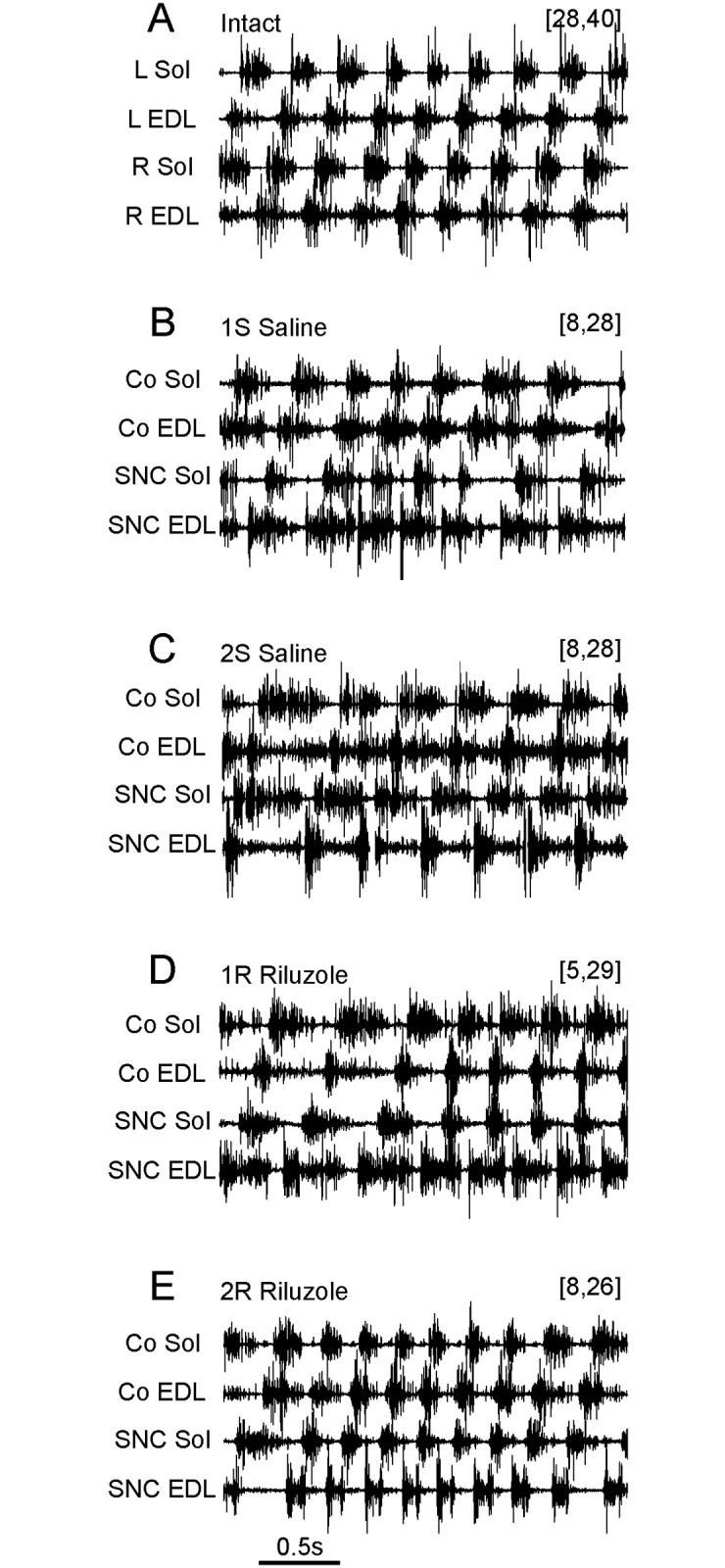
Effects of SNC and treatment with Riluzole on the pattern of EMG activity in representative examples of the experimental groups. (A) Intact rat. (B) Rat from group 1S. (C) Rat from group 2S. (D) Rat from group 1R. (E) Rat from group 2R. The number of motor units in the Sol and EDL muscles are given in brackets. Abbreviations: Sol—soleus, EDL- extensor digitorum longus, L—left, R—right, Co—control, SNC—muscle with SNC.

Observation of locomotor performance indicated that 3 months after SNC locomotor activity of both hind limbs was coordinated and animals walked along the horizontal runway without visible difficulties. However, the toe clearance typical for normal rat plantar walking was missing in the paw of the hind limb with the injured nerve. Visual assessment of bursts of Sol and EDL muscle activity recorded during spontaneous locomotion showed that the general pattern of activity was similar to normal locomotion in that a single burst of EMG activity of the Sol muscle always alternated with a single burst of EDL muscle activity 3 months after SNC in the right hindlimb (Fig 2).

However, visual assessment showed that the duration of bursts of EMG activity of both EDL muscles and Sol-EDL muscle co-activation observed in saline treated rats, with 2–8 (Sol) and 10–28 (EDL) MUs (n = 9) 3 months after SNC, were much longer than those in intact animals (Fig 2A–2C). In contrast, Riluzole treated rats, with 5–16 (Sol) and 15–33 (EDL) MUs after SNC at 1^st^ and 2^nd^ day (n = 13), presented with bursts of EMG activity of both Sol muscles with almost normal duration. Riluzole treated rats, with 5, 6 (Sol) and 15–29 (EDL) MUs (n = 5), showed abnormal prolongation of burst duration of EMG activity of EDL muscles after SNC, while prolongation of burst duration for the control muscle was observed only in one case (Fig 2D). However, the duration of bursts observed in both EDL muscles was reduced in rats with 8–16 (Sol) and 17–33 (EDL) MUs (Fig 2E; n = 8).

The data obtained for Riluzole treated rats with more than 8 MUs in the Sol muscles was not included in further analysis, in order to compare the effects of SNC and treatment with Riluzole rats with the same number of MUs. The effect of treatment on the pattern of activity observed in the EDL muscle depended on the number of MUs in Sol muscles (Table 1, groups 1R and 2R). Thus, the respective data were pooled for further analysis into the RG1 and RG2 (n = 5 and 4) groups with similar numbers of MUs (Table 1).

### Runway locomotion–co-ordination and control of muscle activity

#### Interlimb and intralimb coordination

**Fig 3:**
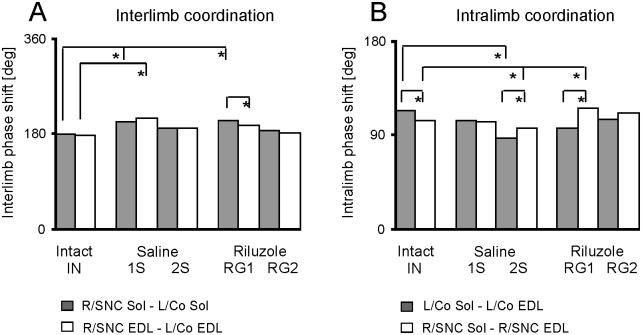
The phase shifts of interlimb and intralimb coordination. (A) Phase shifts for interlimb (R/SNC Sol—L/Co Sol; R/SNC EDL- L/Co EDL) coordination calculated using the cycle duration obtained for the left and control Sol and EDL muscles. (B) Phase shifts for intralimb (L/Co Sol—L/Co EDL; R/SNC Sol—R/SNC EDL) coordination calculated using the cycle durations obtained for the left and right Sol muscles, control Sol muscles and with SNC. Gray and white columns show the mean values obtained in intact, saline and Riluzole treated rats. SEM are not presented in graphical form because they ranged from 0.64 to 1.99%. Abbreviations: L/Co—left/control, R/SNC—right/muscle with SNC, Sol—soleus, EDL- extensor digitorum longus, *—*p <* 0.001.

In intact rats the phase shifts of interlimb coordination attained similar values of 178 ± 14 and 175 ± 26 deg for coordination established, based on left compared to right Sol and EDL muscles (Fig 3A; S1 Table).

Both saline (1S and 2S) and Riluzole (RG1 and RG2) treated rats showed modest increases in phase shifts compared to those in intact rats. However, the Kruskal-Wallis test (H_(9, 2100)_ = 172.425, *p <* 0.001) and multiple comparison test showed that only the increase (13 to 19%) found in the 1S rats for both muscles and in the RG1 rats for the Sol muscles were significant (*p* < 0.001). The above analysis showed also that only phase shifts obtained in the RG1 rats established based on left *vs*. right Sol muscles differed (*p* < 0.001) from that established based on left *vs*. right EDL muscles (Fig 3A).

The phase shifts of intralimb coordination (flexor *vs*. extensor muscles) in intact rats which differed (*p* < 0.001) slightly (10%) between sides were 113 ± 20 and 103 ± 16 deg for the left and right side muscles (Fig 3B; S1 Table). Both saline (1S and 2S) and Riluzole (RG1 and RG2) treated rats manifested modest alterations in phase shifts compared to intact rats. The decreases (24 and 7%) in the 2S rats and the increase (15%) in the RG1 rats for muscles with SNC were significant (H_(9, 2100)_ = 1768,198, *p <* 0.001 and *p* < 0.001; (Fig 3B). In addition the phase shifts obtained for intralimb coordination differed (*p* < 0.001) significantly (9 to 23%) between the left and right side muscles between SNC and in intact animals as well as between saline and Riluzole treated rats. However, phase shifts of inter- and intralimb coordination in the RG2 rats were not significantly different (*p* = 1.0) from intact rats (Fig 3A and 3B).

The strength of inter- and intralimb coordination estimated with the length of vector ***r*** was highly significant (*p* < 0.001) in all groups and attained values of 0.95–0.98 in intact rats, while saline and Riluzole treated rats showed ranges of 0.84–0.98 and 0.79–0.97, respectively. Kruskal-Wallis tests (H_(9, N = 42)_ = 28.265 and 13.473, *p*: < 0.001 and 0.142) and multiple comparison tests (*p*: 0.446–1.0 and 0.660–1.0) showed that strengths of inter- and intralimb coordination did not differ between any experimental groups. Neither SNC, nor treatment with Riluzole affected the strength of inter- and intralimb coordination despite some differences in phase shifts between experimental groups.

#### The duration of cycle

**Fig 4:**
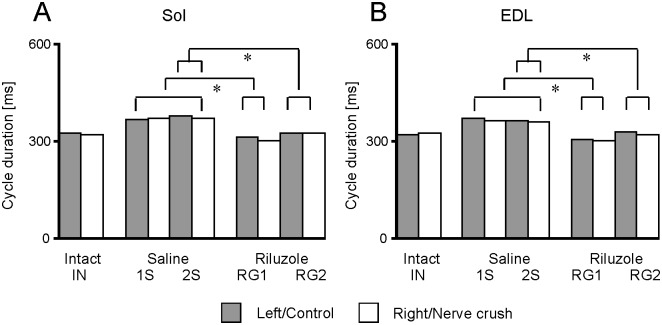
Changes in the cycle duration induced by SNC and treatment with Riluzole. Gray and white columns show the means of cycles established based on the Sol (A) and EDL (B) muscles for the left and right muscles as well as for control muscles and muscles with SNC in intact, saline and Riluzole treated animals. The values of SEM are not presented in graphical form because they were from 1.78 to 3.20%. Abbreviations: *—*p* < 0.005.

In intact rats the cycle durations ranging from 320 ± 7 to 322 ± 73 ms were not significantly different (Kruskal-Wallis tests, H_(9, 2100)_ = 113.117 and 108.395, *p* < 0.001 and multiple comparisons tests, *p* = 1.0) between the left and right muscles and between Sol and EDL muscles (Fig 4; S2 Table).

Similarly, the differences between the cycle durations obtained for the control muscles and muscles with SNC in saline and in Riluzole treated rats (0.2–3.0%, *p* = 1.0) were not significant. Comparison of cycle durations between experimental groups showed that in Riluzole treated RG1 rats durations were significantly shorter (15 to 18%, *p* < 0.005) than in saline treated animals (1S and 2S) and those in the RG2 rats were significantly shorter (8 to 12%, *p* < 0.002) when compared with the 2S rats. However, despite this difference, the cycle durations in saline treated rats were not significantly longer (11 to 15%, *p*: 0.654–1.0), whereas those in Riluzole treated rats were not significantly different and changed by -7 to 3% (*p*: 0.071–1.0) compared with those in intact rats.

#### The relationship between the burst and cycle duration of EMG activity for Sol and EDL muscles

**Fig 5:**
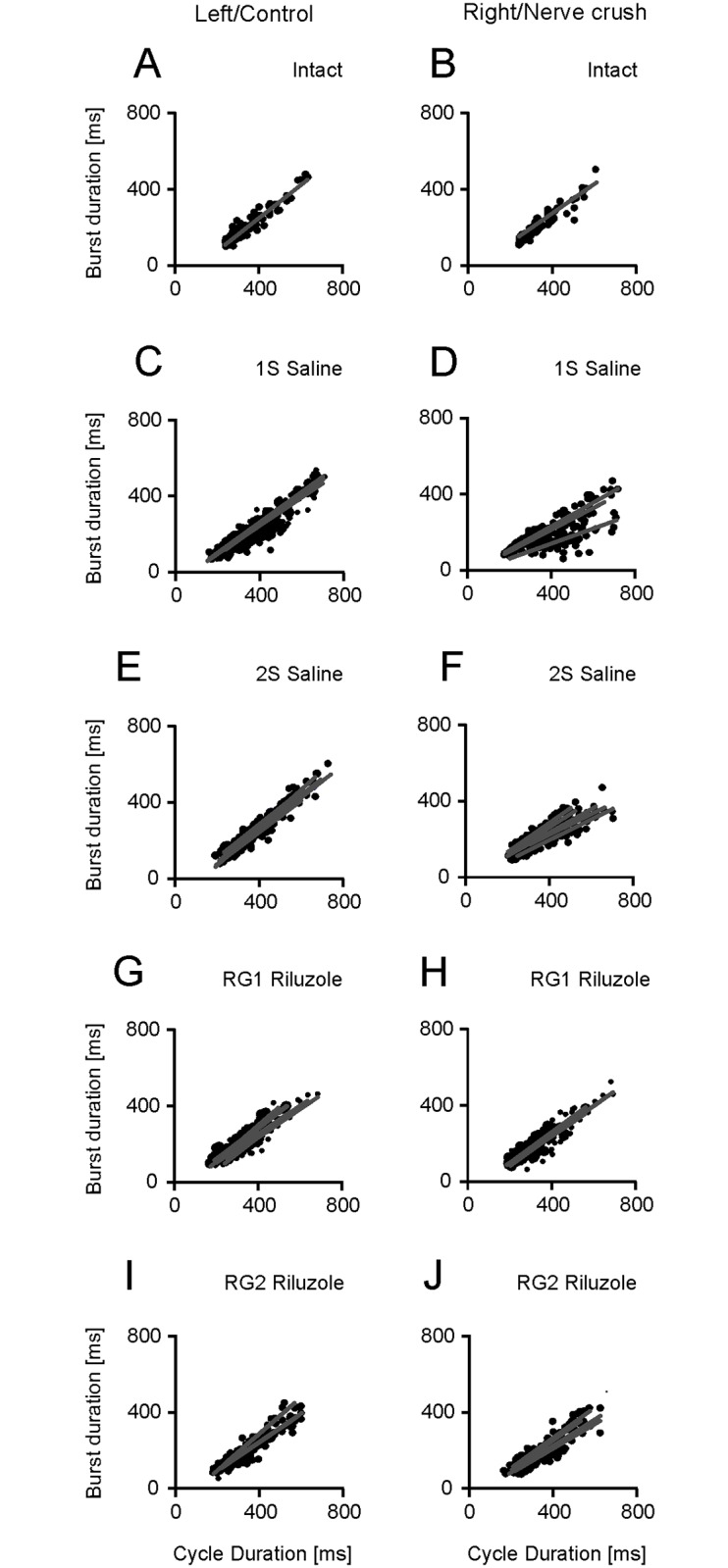
Effects of Riluzole treatment after SNC on the relationships for the Sol muscles. (A-J) Regression lines obtained for the left and right muscles in individual intact rats, control muscles and muscles with SNC in saline and Riluzole treated rats.

In intact rats the relationship between burst durations for Sol muscle *vs*. step cycle durations were significantly different (*p* < 0.001) with correlation coefficients of 0.927–0.960 in both hindlimbs. The regression slopes and intercepts ranged from 0.82 to 0.89 and from -91 to -76 ms (Fig 5A and 5B; S3 Table).

The saline (1S and 2S) and Riluzole treated rats (RG1 and RG2) showed correlation coefficients ranging from 0.648 to 0.995 and from 0.885 to 0.972 (*p* < 0.001) indicating that the relationship between burst duration and step cycle duration remained significant in both saline and Riluzole treated rats (Fig 5C–5J; S3 Table). The relationship between the burst duration (*T*_BD_) and the cycle duration (*T*_C_) was described by linear regression *T*_BD_ = *aT*_C_ + *b*, where: *a* and *b* denote the slope and intercept of the regression line. The slope and intercept of regression lines obtained in saline treated rats (1S and 2S) for control Sol muscles remained significant (*p* < 0.001) with values ranging from 0.74 to 0.99 and from -129 to -49 ms indicating a small effect of SNC. The slope and intercept for muscles with SNC inflicted at 1^st^ day a.b. attained values from 0.38 to 0.61 (*p* < 0.001) and from -16 to -3 ms (*p*: 0.161–0.878), while those for muscles with SNC at 2^nd^ day a.b. (2S) were 0.53 to 0.76 (*p* < 0.001) and -16 to -2 ms (*p*: 0.126–0.972) (Fig 5C–5F; S3 Table). The slope decreased by 9–46%, while the intercept increased significantly. Linear regression *T*_BD_ = *aT*_C_ + *b* revealed that the change in the burst duration for muscle with SNC following a change in the cycle duration was shorter than the change in the burst duration for the control muscle. The effect of a decrease in the regression slope on the burst duration was not compensated by the increase in the intercept, which indicated a reduction in the duration of burst for Sol muscle with SNC compared to that of control muscle. This effect was observed mainly in rats with SNC at 1^st^ day a.b. and showed after SNC there was impaired adaptation of Sol muscle activity duration to both the cycle and control muscle activity durations (Fig 5C and 5D; S3 and S5 Tables).

In Riluzole treated rats (RG1 and RG2) the slope of regression lines for Sol muscles with SNC was increased, while the intercept was decreased when compared with the values obtained in saline treated rats (1S and 2S). The, change in slope and intercept for the muscles with SNC was highly significant (*p* < 0.001) ranging from 0.63 to 0.80 and from -70 to -46 ms (Fig 5G–5J; S3 Table) indicating Riluzole induced recovery.

**Fig 6:**
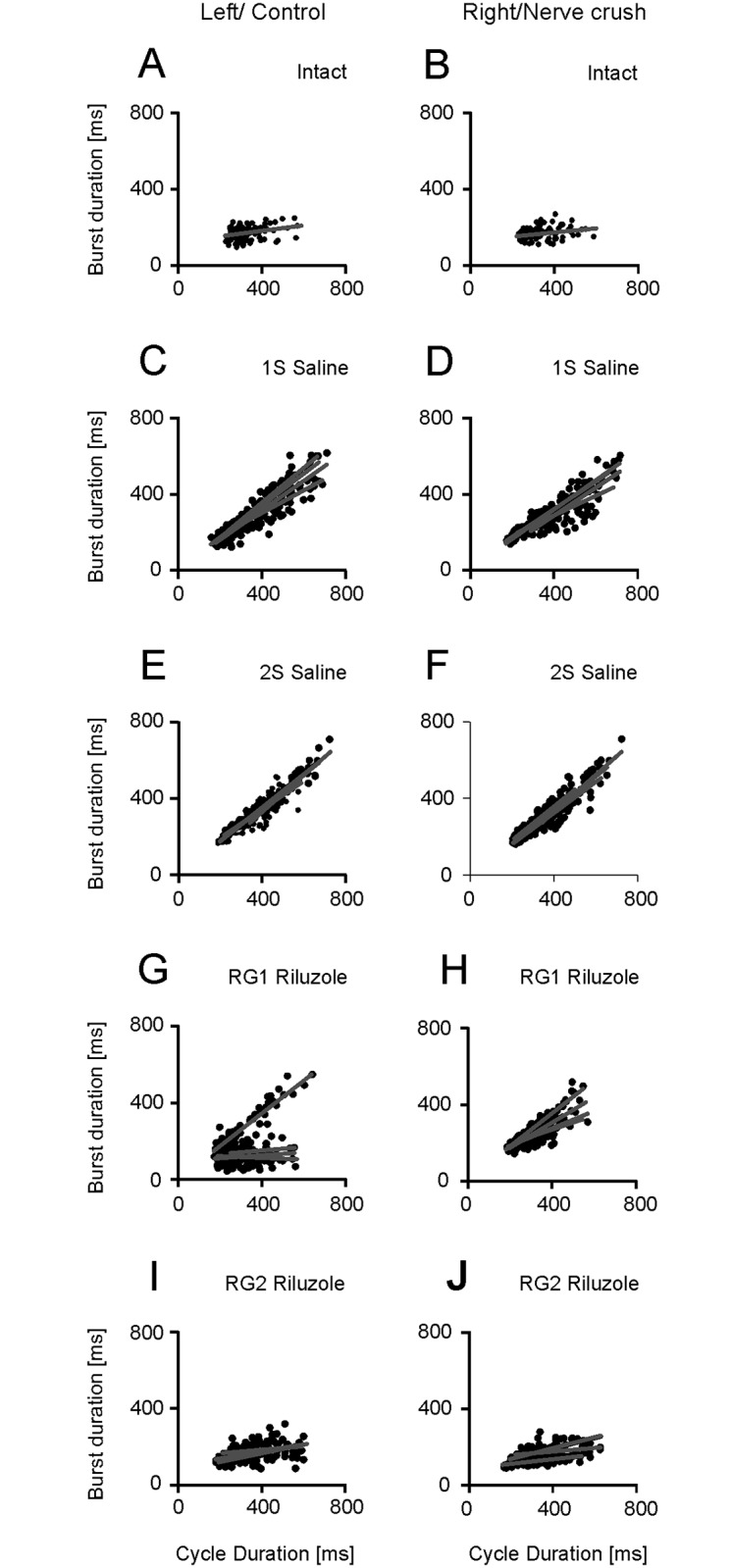
Effects of Riluzole treatment after SNC on EDL muscles. (A-J) Regression lines obtained for left and right muscles in individual intact rats, control muscles and muscles with SNC in saline and Riluzole treated rats.

In contrast to Sol muscles EDL muscles in intact rats showed small correlation coefficients of 0.138–0.445 (*p*: 0.001–0.339) and flat linear regression slopes of 0.06–0.21 accompanied by a significant (*p* < 0.001) increase in positive intercepts ranging from 99 to 158 ms (Fig 6A and 6B; S3 Table).

In contrast, in saline treated rats (1S and 2S) the change in relationships for control EDL muscles and EDL muscles with SNC was highly significant (*p* < 0.001) with correlation coefficients of 0.792 to 0.964. The increase in the correlation coefficients was accompanied by a systematic increase in the slope of regression and a decrease in the intercept, which attained values of 0.59 to 0.92 and of -15 to 73 ms (*p*: 0.015–0.936) (Fig 6C–6F; S4 Table). The increase in the slope for the EDL muscles in saline treated rats in contrast to Sol muscles indicated that the adaptation of flexor muscle activity duration to the duration of the cycle was similar to that for the extensor muscle. Such an observation confirms a serious deficit in the process of controlling locomotor movement because it prevents essential increases or decreases in the duration of flexor activity according to the change in cycle duration.

The effect of treatment with Riluzole on EDL muscle EMG burst activity in contrast to the Sol muscle differed between rats with different numbers of MUs. In RG1 rats (n = 5) with 5, 6 (Sol) and 15–29 (EDL) MUs four of them showed almost normal relationships for control muscles, whereas the remaining relationships for control muscles and for muscles with SNC were similar to those in saline treated rats (Fig 6G and 6H; S4 Table). Whereas, Riluzole treated RG2 rats with 8 (Sol) and 23–33 (EDL) MUs showed a slight difference in the slope and intercept of regression lines with values of 0.02–0.26 and 85–141 ms (*p* < 0.001) as compared to those in intact rats, while the correlation coefficients of 0.192–0.749 (*p*: < 0.001–0.181) were greater than those in intact rats (Fig 6I and 6J; S4 Table).

The described analysis showed that SNC did not impair the relationships observed for Sol muscles, but induced changes which diminished the adaptation of burst duration to the cycle duration. Whereas, the relationships for EDL muscles became similar to those for the extensor muscles. Treatment with Riluzole counteracted the effect of SNC and restored almost normal relationships for both the Sol muscles in rats (RG1) with 5–8 and 15–33 MUs (n = 9) and EDL muscles in rats (RG2) with 8 and 23–33 MUs (n = 4).

#### The duration of burst and the duty factor of Sol muscle EMG activity

**Fig 7:**
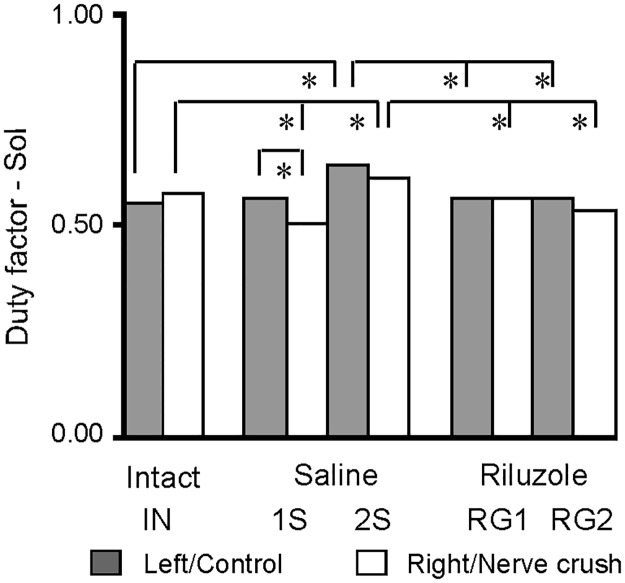
Effects of Riluzole treatment after SNC on the duration and duty factor of burst for the Sol muscles. Gray and white columns show the means for the left and right muscles in intact rats, control muscles and muscles with SNC, in saline and Riluzole treated rats. The values of SEM are not presented in graphical form because they were very small (from 0.71 to 1.67%). Abbreviations: *—*p* < 0.011.

Analysis of the duty factor (relative duration) was used to characterize the effects of SNC and treatment on the structure of the cycle and changes in the relationship between the burst and cycle duration through observation of changes in the relative duration of EMG activity. In intact rats burst durations of 181 ± 62 and 196 ± 66 ms were found for the left and right Sol muscles, which corresponded to duty factors of 0.55 ± 0.07 and 0.57 ± 0.08 differing slightly (*p* = 0.011) between muscles (Fig 7; S5 Table).

In saline treated rats (1S and 2S) the burst duration and duty factors ranged from 180 ± 80 to 236 ± 95 ms (0.50 ± 0.11 to 0.64 ± 0.09), whereas in Riluzole treated rats (RG1 and RG2) they were 167 ± 63 to 189 ± 81 ms (0.55 ± 0.09 to 0.56 ± 0.10) (Fig 7; S5 Table). In intact and Riluzole treated rats both indices were within a narrow range of 55–57% of cycle duration. On the other hand in saline treated animals they occupied 50–64% of the cycle. Data obtained for intact and saline treated rats showed that SNC inflicted at 1^st^ day a.b. induced a significant decrease (12%; (*p* < 0.001)) in the duty factor for muscles with SNC, while in the remaining instance both duty factors were increased (*p*: < 0.001 and 0.007) slightly (6–16%). However, both duty factors obtained for Riluzole treated rats (RG1 and RG2) did not differ (*p* = 1.0) from those in intact rats (Fig 7; S5 Table).

The effects of SNC on the burst duration and the duty factors of Sol muscle EMG activity were small but statistically significant and consistent with the effects on the relationship between the burst and cycle duration observed; providing evidence that changes in the parameters of these relationships reflected small but significant deficits in muscle control.

#### The duration of burst and the duty factor of EDL muscle EMG activity

**Fig 8:**
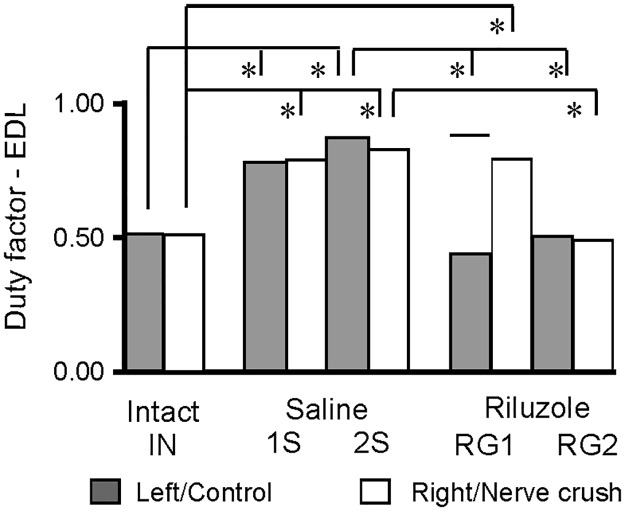
Effects of Riluzole treatment after SNC on the duty factor of burst for EDL muscles. Gray and white columns show the mean for the left and right muscles in intact rats, control muscles and muscles with SNC, in saline and Riluzole treated rats. Additional horizontal bar in the RG1 group shows the mean in rat RB5 which did not develop normal activity in the control muscle. The values of SEM are not presented in graphical form because they were from 0.57 to 3.96%. Abbreviations: *—*p* < 0.001.

In intact rats burst durations of 165 ± 32 and 165 ± 30 ms and duty factors of 0.51 ± 0.12 and 0.52 ± 0.11 did not differ (*p* = 1.0) between left and right EDL muscles (Fig 8; S6 Table).

In rats treated with saline (1S and 2S) SNC led to a highly significant (*p* < 0.001) increase in the burst duration even though the duration of the cycle was not significantly increased. The burst durations approached values of 287 ± 112 to 317 ± 93 ms (0.78 ± 0.11 to 0.87 ± 0.07) (Fig 8; S6 Table). It can be demonstrated that the prolongation of EDL muscle EMG activity found in all our saline treated rats, when changes in the burst duration of Sol muscle activity and changes in the coordination of Sol and EDL muscle activity are small, leads to prolonged co-activation of these muscles.

In Riluzole treated rats the burst duration of EDL muscle activity depended on the number of MUs rescued in their Sol and EDL muscles. Burst duration of 133 ± 88 ms (0.43 ± 0.22) was obtained for control muscles in four RG1 rats and 282 ± 107 ms (0.87 ± 0.10) in fifth of them, consistent with the effect of treatment on the respective relationships and the lack of such effect in the remaining case (Fig 8; S6 Table). However, the duration of bursts obtained for the muscles with SNC was increased to 238 ± 64 ms (0.79 ± 10) consistent with the lack of effect of treatment on the relationships observed (Fig 8 and S6 Table). However, Riluzole treated RG2 rats (n = 4) with 8 and 23–33 MUs showed burst durations of 161 ± 45 and 154 ± 36 (0.50 ± 0.12 and 0.49 ± 0.11) with no significant (*p* = 1.0) difference between muscles and no significant (*p* = 1.0) difference compared with those in intact rats consistent previous observations (Fig 8; S6 Table). The significant effect of SNC on the burst duration and the duty factor of EDL muscle activity provided additional evidence that changes in the parameters of these relationships reflected severe and significant deficits in muscle control.

The effects of treatment with Riluzole on the burst duration and the duty factor for the EDL muscles generally depended on the number of MUs. However, rats from a subgroup (n = 3) of saline treated animals (Table 1) did not recover EDL muscle EMG activity with normal burst durations and duty factors even if the numbers of MUs were 8 (Sol) and 24–28 (EDL). On the other hand, rats from subgroup (n = 3) of Riluzole treated animals (Table 1) with similar numbers of MUs, 8 (Sol) and 23–26 (EDL), showed almost normal activity in both muscles (Table 1; Fig 8; S6 Table). The relationship between the effect of SNC, treatment with Riluzole and the number of MUs supports the suggestion that the number of MUs was not the only determinant of treatment effectiveness and that SNC and treatment with Riluzole also affected the neuronal circuits controlling locomotion.

#### The relationships between the duty factors established for right and left hindlimb (with SNC and control)

**Fig 9:**
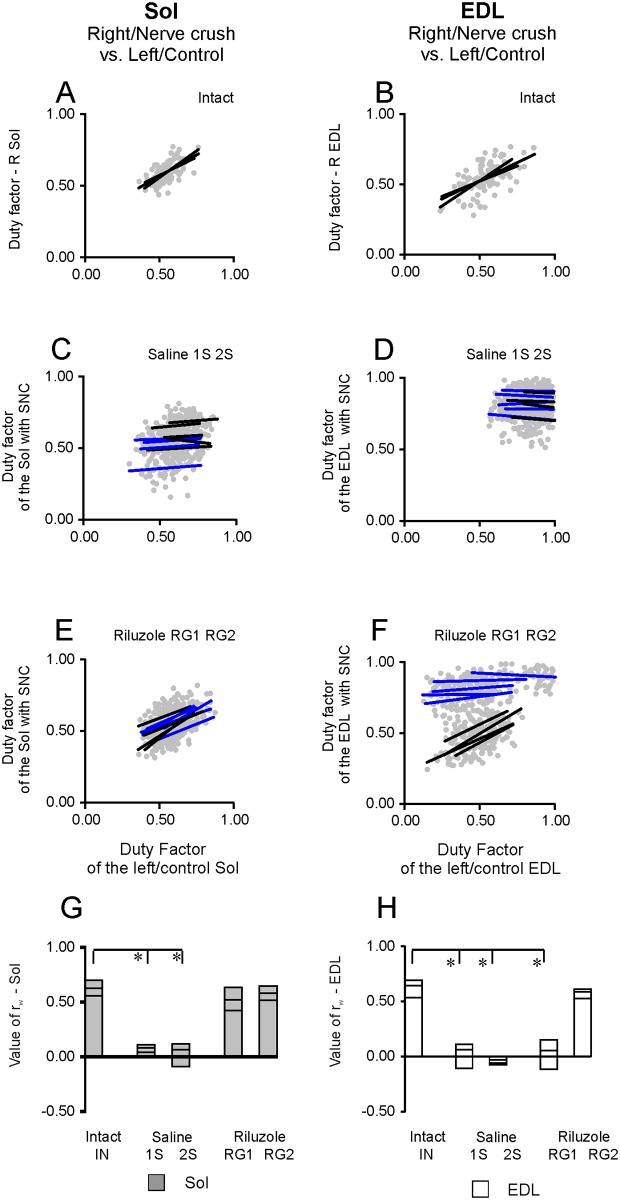
The relationship between the duty factors established for right and left hindlimb (with SNC and control). (A-D) Regression lines for the relationships observed in right and left muscles in individual intact animals and for muscles with SNC and control muscles in rats treated with saline after SNC at the 1^st^ day a.b. (1S, blue) and the 2^nd^ day (2S, black). (E, F) Regression lines for the relationships observed for the muscles with SNC and control muscles in individual Riluzole treated rats with 5, 6 and 15–29 MUs (RG1, blue) and in rats with 8 and 23–33 MUs in Sol and EDL muscles (RG2, black). (G, H) Gray and white columns and lower horizontal bars within columns show maximum and minimum values of the correlation coefficients for Sol and EDL muscles in individual rats, whereas upper bars within a column show the values of common correlation coefficients in intact, saline and Riluzole treated rats. Abbreviations: R-right, SNC-sciatic nerve crush, *r*_w_-common correlation coefficient,*—*p* < 0.001.

In individual intact rats the relationship between the duty factors for both Sol and EDL muscles showed significant (*p* < 0.001) correlation coefficients of 0.558–0.699 and 0.534–0.690 with regression line slopes of 0.506–0.818 indicating that all analyzed relationships were significant and strong (Fig 9A and 9B; S7 Table). Tests for the heterogeneity of correlation coefficients obtained both for the Sol and EDL muscles in individual rats (chi^2^ = 1.41 and 1.54, df = 2) showed that they came from populations having identical rho’s (*p*_w_:*0*.527 and 0.463) and so did not differ between individual rats. The common correlation coefficient *r*_w_ of 0.640 (chi^2^ = 2.95, df = 5) obtained for both the Sol and the EDL muscles (*p*_w_ = 0.707) showed that the strength of the respective relationship did not differ between muscles (Fig 9G and 9H; S7 Table).

In contrast, all our rats treated with saline (n = 9) showed small linear regression slopes and no significant correlation from the ranges of -0.101 to 0.097 and -0.110 to 0.117 (*p*: 0.418–0.944) for both Sol and EDL muscles. Tests for the heterogeneity (chi^2^: 0.071.69, df = 3 and 4) showed that the common correlation coefficients *r*_w_ for rats treated with saline after SNC at the 1^st^ and the 2^nd^ (1S and 2S) day were 0.092 and 0.059 (*p*_w_: 0.988 and 0.792) for Sol muscles and 0.015 and -0.062 (*p*_w_: 0.760 and 0.999) for EDL muscles (Fig 9G and 9H; S7 Table). Similar analysis (chi^2^ = 1.56 and 1.88, df = 8) showed that the correlation coefficients obtained for saline treated rats (1S and 2S) did not differ between individual rats both for Sol and EDL muscles (*p*_w_: 0.991 and 0.984) with common correlation coefficients *r*_w_ which were 0.072 and -0.028. Further, comparison of correlation coefficients obtained for both Sol and EDL muscles in intact and in saline treated rats enabled us to reject (chi^2^ = 48.59 and 64.81, df = 11, *p* < 0.001) the hypothesis that they came from populations having identical rho’s (Fig 9G and 9H; S7 Table). This analysis showed that SNC disrupted the correlation between the duty factor of burst for muscle with SNC and the duty factor of burst for control muscle for both Sol and EDL muscles (Fig 9C, 9D, 9G and 9H; S7 Table).

In contrast to saline treated animals all our Riluzole treated rats (n = 9) developed activity of Sol muscles with significant correlation between their relative durations. The correlation coefficients and slopes were within ranges of 0.424–0.647 and 0.346–0.844 (*p*: < 0.001–0.002), while the common coefficient of correlation *r*_w_ was 0.550 (chi^2^ = 4.73, df = 8, *p* = 0.786) (Fig 9E and 9G; S7 Table). The correlation coefficients obtained for all Riluzole treated rats did not show the heterogeneity when compared with those for intact rats (chi^2^ = 7.42, df = 11, *p* = 0.764). This indicated that treatment with Riluzole enabled almost normal correlation between duty factors for Sol muscles. However, the effect of treatment with Riluzole on the EMG activity of EDL muscles depended on the number of rescued MUs. The relationship between the duty factors found in the RG1 rats with 5,6 and 15–29 MUs was not significant with the correlation coefficient and slopes of -0.118 to 0.148 (*p*: 0.304–0.944) and -0.056 to 0.141, while *r*_w_ was 0.051 (chi^2^ = 2.32, df = 4, *p* = 0.678) (Fig 9F and 9H; S7 Table). In addition, comparison with data obtained in intact rats (chi^2^ = 57.51, df = 7, *p* < 0.001) showed that they did not come from populations with identical values of rho. On the other hand the RG2 rats with 8 (Sol) and 23–33 (EDL) MUs showed much greater and significant values of 0.526–0.611 (*p* < 0.001) and 0.449–0.632, while *r*_w_ was 0.585 (chi^2^ = 0.49, df = 3, *p* = 0.921) (Fig 9F and 9H; S7 Table). Consistent with the values of *r* obtained for intact and RG2 rats with 8 (Sol) and 23–33 (EDL) MUs they came from populations having identical rho’s (chi^2^ = 2.35, df = 6, *p* = 0.884), indicating that treatment with Riluzole enabled almost normal correlation between the duty factors of EMG bursts for EDL muscles.

Taken together, our analysis provided evidence that SNC disrupted the correlation between the activity of both Sol and EDL muscles in all saline treated rats (Fig 9C–9D, 9G and 9H; S7 Table). The effect of SNC did not depend on the age of our rats at the time of SNC or the numbers of MUs in their Sol and EDL muscles (2–8 and 10–28, respectively). Riluzole treated RG1 rats (n = 5) with 5, 6 (Sol) and 15–29 (EDL) MUs as well as RG2 animals (n = 4) with 8 (Sol) and 23–33 (EDL) MUs developed EMG activity of Sol muscles with almost normal correlation. However, rats from a subgroup (Table 1) of saline treated animals (n = 5) with 6, 8 (Sol) and 10–28 (EDL) MUs showed a lack of correlation. Moreover, rats from a subgroup (Table 1) of saline treated animals (n = 3) with 8 (Sol) and 24–28 (EDL) MUs did not develop normal correlations for EDL muscles, whereas a subgroup of Riluzole treated RG2 rats (n = 3) of these animals (Table 1) with almost the same numbers of MUs, 8 (Sol) and 23–26 (EDL), showed activity of EDL muscles with a strong correlation between the duty factors (Fig 9C–9H; S7 Table).

The results described above support our previous suggestion that the number of MUs [5] was not the only determinant of treatment effectiveness and that treatment with Riluzole restored normal EMG activity of both Sol and EDL muscles due to an action on the number of MUs and the neuronal circuit involved in locomotor control. The lack of correlation between the duty factor for muscles with SNC and for control muscles found in saline treated rats is a serious deficit in locomotor movement control. It precludes or attenuates the appropriate adaptation of muscle activity observed as an increase or decrease in the duty factor of burst for muscles with SNC according to a change in the duty factor of burst for the control muscles, which is a prerequisite for locomotor movement.

#### The relationships between the burst duration of EDL and Sol muscle EMG activity

**Fig 10:**
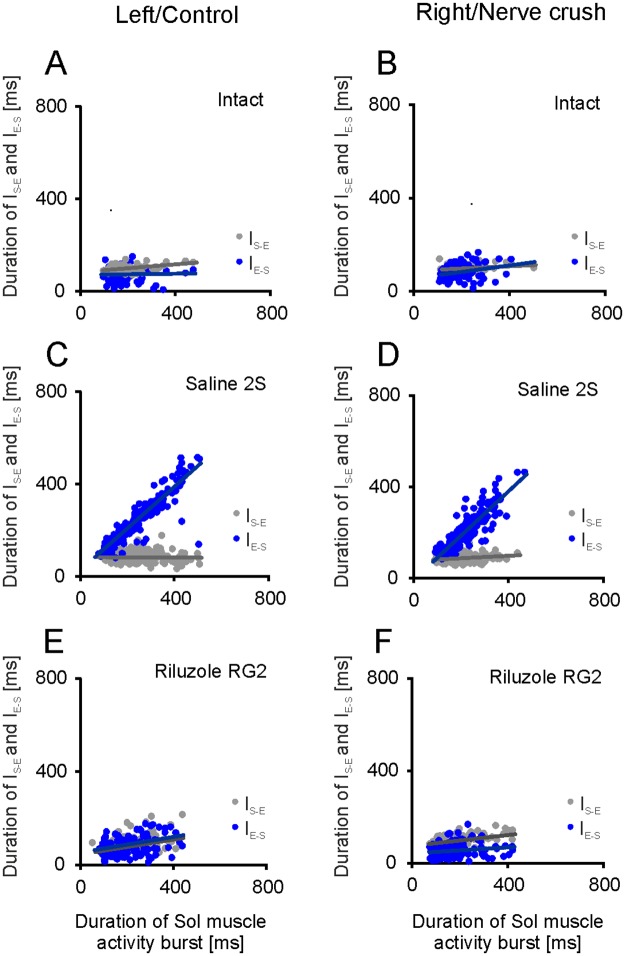
The relationships between the durations of I_S-E,_ and I_E-S_ intervals and burst of Sol muscle EMG activity. (A-D) Regression lines for the relationships obtained for left and right muscles in intact rats and for control muscles and muscles with SNC in rats treated with saline after SNC at 2^nd^ day a.b. (2S). (E, F) Regression lines for the relationships observed for control muscles and muscles with SNC in Riluzole treated rats with 8 and 23–33 MUs in Sol and EDL muscles (RG2).

The data in Fig 10 show the relationship between the duration of the I_S-E_ and I_E-S_ intervals (Fig 1) with the burst duration of Sol muscle EMG activity obtained for left and right muscles in intact rats as well as control muscles and SNC muscles in saline and Riluzole treated rats.

In all rats the slopes of regression and the coefficient of correlation for I_S-E_ intervals for both hindlimb muscles were small within a ranges of 0.008–0.184 and 0.110–0.597 (*p*: < 0.001–0.820) in all instances except one case with a value of 0.033 (Fig 10A–10F; S8 and S9 Tables). The correlation coefficients obtained for saline (chi^2^ = 11.93 and 6.04, df = 11, *p*: 0.368 and 0.870) and Riluzole treated rats (chi^2^ = 11.05 and 11.41, df = 11, *p*: 0.439 and 0.409) did not differ from those in intact rats. The duration of the I_S-E_ interval in intact rats was 91 ± 14 and 86 ± 11 ms for the left and right muscles, while in saline and Riluzole treated rats they varied from 78 ± 18 to 98 ± 29 ms (S8 and S9 Tables). The interval durations obtained in saline and Riluzole treated rats differed from those in intact animals by -3 to 7 ms, except for differences of -8 and -13 ms for control muscles in the 2S and RG1 rats. Kruskal-Wallis test (H_(9, 2100)_ = 144.233, *p* < 0.001) and test for multiple comparisons showed that the interval durations did not differ significantly between the left and right muscles (*p*: 0.282–1.0) and from those in intact rats (*p*: 0.886–1.0), except for the 2S and RG1 rats. The predicted duration of the I_S-E_ intervals in saline and Riluzole treated rats ranging from 78 to 94 ms did not differ significantly (H_(9, 2100)_ = 157.542, *p* < 0.001 and *p*: 0.108–1.0) from the values in intact rats, except for those in the 2S and RG1 rats (S8 and S9 Tables).

**Fig 11:**
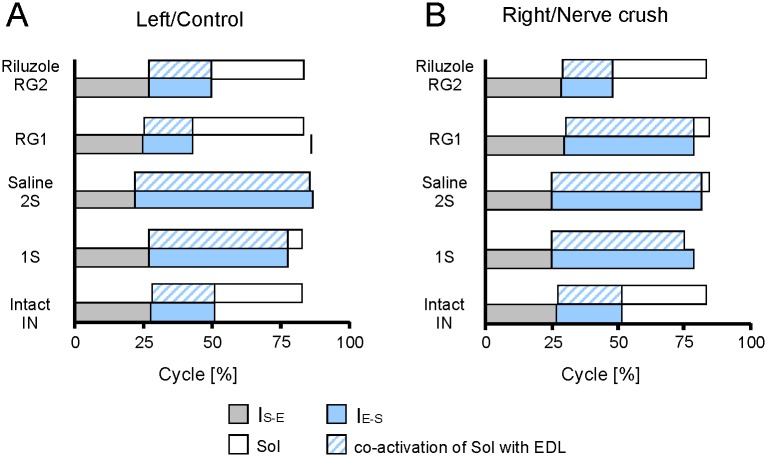
Effects of Riluzole treatment after SNC on the burst duration of EDL muscle EMG activity and co-activation with Sol muscle. (A, B) Horizontal bars show the duration of burst of Sol and EDL muscle EMG activity presented as the sum of I_S-E_ and I_E-S_ interval durations, with the duration of muscle co-activation expressed as a percentage of cycle duration, for left and right muscles in intact rats, control muscles and muscles with SNC, in saline and Riluzole treated rats. Additional horizontal bar in the RG1 group shows the mean duration in rat RB5 which did not develop normal activity in the control muscle.

Taken together, these results provide evidence that both in saline and Riluzole treated rats the I_S-E_ interval duration was similar within the range of 22 to 30% of cycle duration (Fig 11, S8 and S9 Tables). In addition the results showed that the effect of SNC on the EDL muscle burst durations through action on the I_S-E_ interval duration (-4.0 to 2.5% of cycle duration) was not significant or small (S8 and S9 Tables). The lack of effect of SNC on the relationship described and the durations of I_S-E_ time intervals implies that the prolongation of EMG activity of EDL muscles results in the prolongation of co-activation of Sol and EDL muscles.

The relationships analyzed for the I_E-S_ intervals showed a much stronger effect of SNC. The slope of regression and coefficient of correlation obtained for I_E-S_ intervals was increased to 0.680–1.142 and 0.844–0.975 (*p* < 0.001) in saline treated rats (1S and 2S), while in intact rats they were 0.062–0.234 (*p*: 0.004–0.135) and 0.214–0.399 (Fig 10A–10D; S8 and S9 Tables). The correlation coefficients obtained for intact and saline treated rats were strongly (chi^2^ = 263.45 and 175.16, df = 11, *p* < 0.001) heterogeneous independent of the number of MUs that survived in the Sol and EDL muscles (2–8 and 10–28). Whereas, the effect of SNC observed in Riluzole treated rats depended on the number of MUs. In Riluzole treated RG1 rats with 5, 6 (Sol) and 15–29 (EDL) MUs four of five animals showed the relationships for control muscles with the slopes of regression and correlation coefficients similar (chi^2^ = 3.23, df = 6, *p* = 0.779) to those in intact rats, whereas the relationships observed in the remaining instances were similar to saline treated rats. However, Riluzole treated RG2 rats with 8 (Sol) and 23–33 (EDL) MUs showed small correlation coefficients and regression slopes of 0.134–0.386 (*p*: < 0.005–0.353) and 0.025–0.108 (Fig 9E–9F; S8 and S9 Tables). The correlation coefficients obtained in intact rats and in RG2 rats showed (chi^2^ = 2.67 and 2.59, df = 6, *p*: 0.848 and 0.858) they came from populations with identical values of rho.

The duration of both I_E-S_ intervals were strongly affected by SNC consistent with the effect on the respective relationships. In saline treated rats (S1 and S2 Tables) the durations of 188 ± 95 to 241 ± 100 ms were (H_(9, 2100)_ = 1266.933, *p* < 0.001) significantly (*p* < 0.001) increased by 117–171 ms compared with the values of 70 ± 28 and 81 ± 30 ms for the left and right muscles in intact rats (S8 and S9 Tables). In Riluzole treated RG1 rats with 5, 6 (Sol) and 15–29 (EDL) MUs the duration of 52 ± 23 ms was not different (*p* = 0.464) from that in intact rats was obtained for control muscles in four of five animals, whereas in the remaining instance it was 187 ± 92 ms. Whereas, the duration of 147 ± 46 ms obtained for muscles with SNC remained much longer (*p* < 0.001) compared with that in intact rats. In contrast, in Riluzole treated RG2 rats with 8 and 23–33 MUs both durations were reduced to 72 ± 35 and 59 ± 20 ms. The duration of interval for control muscles showed no significant difference (*p* = 1.0) from that in intact rats, while the duration for muscles with SNC was shorter (*p* = 0.034) by 22 ms (S8 and S9 Tables). The difference between the predicted duration of I_E-S_ intervals obtained in saline treated rats and the duration in intact animals ranging from 92 to 130 ms, as well as the difference of 120 ms obtained for muscles with SNC in the RG1 rats were (H_(9, 2100)_ = 1105.548, *p*<0.001) highly significant (*p*<0.001). On the other hand, the difference of -18 ms (*p* = 0.313) obtained for control muscles in four of five RG1 rats and the difference of 1 ms (*p* = 1.0) for control muscles in the RG2 rats was not significant, while the difference of -21 ms (*p* = 0.026) obtained for muscles with SNC in the RG2 rats was almost significant (S8 and S9 Tables). The data described suggests SNC induced dramatic increases in the duration of both EDL muscle EMG activity and co-activations with Sol muscles. In rats treated with Riluzole this effect was significant for muscles with SNC in the RG1 group of rats. In contrast, the RG2 rats showed small or no significant effect of SNC both for control muscles and muscles with SNC showing that treatment with Riluzole enabled recovery of almost normal EMG activity of both EDL muscles in rats with 8 MUs in Sol muscles.

In saline treated rats the contribution of the I_E-S_ interval durations to the burst durations of EDL muscle EMG activity was 51 to 65% of cycle duration. On the other hand, in intact and Riluzole treated RG2 rats this contribution was 19 to 25% of cycle duration (Fig 11, S8 and S9 Tables). In saline treated rats the increase in the burst durations of EDL muscle EMG activity due to the effect of SNC on the interval durations, ranged from 28 to 38% of cycle duration. In contrast in Riluzole RG2 rats they were 0.6 and -6% of cycle duration. Similarly the predicted duration showed that the effect of SNC on the burst durations through action on the respective relationships approached 25 to 33% of the cycle in saline treated rats, whereas in Riluzole treated RG2 rats it was 0.3 and -6% of the cycle duration. Our analysis showed that the relative duration of co-activation of Sol and EDL muscle activity observed in rats treated with saline corresponded to 50–64% of cycle, whereas in intact and Riluzole treated RG2 rats they were 19–25% of cycle duration (Fig 11, S8 and S9 Tables).

In summary, the burst duration of EDL muscle EMG activity was not affected by SNC through an action on the I_S-E_ interval. In contrast, the effect of SNC through an action on the I_E-S_ interval was strong and similar for both muscles in saline treated rats. In all instances this effect manifested as a prolongation of EDL muscle activity burst after the onset of Sol muscle activity. The prolongation of EDL muscle EMG activity in the absence of an essential effect of SNC on the time interval between the onset of EMG activity in the Sol and EDL muscles, prolonged their co-activation, which in turn diminished forces produced by hindlimbs during both stance phases of the cycle. A similar effect was found for muscles with SNC in the RG1 rats with 5, 6 MUs rescued in their Sol muscles. In contrast, the RG2 rats with no fewer than 8 MUs developed normal EMG activity of both EDL muscles with attenuation of the EMG activity during the stance phase and reduced duration of flexor-extensor co-activation. The relationship between the effect of treatment and the number of MUs does not imply that the number of MUs was the only determinant of recovery, because saline treated rats with the same number of MUs showed EMG activity in both EDL muscles with abnormal burst duration. This suggests that abnormal control of EDL muscles in rats with SNC can be explained by malfunction of neuronal circuits which “program” inhibition of EDL muscle activity during the stance phase with the contribution of sensory information from the Sol muscle.

## Discussion

In this study we analyzed for the first time the effects of SNC in new born rats and treatment with Riluzole on soleus and extensor digitorum longus muscle activity during locomotion. Using EMG activity recorded simultaneously from both extensor and flexor muscles of the ankle joint of both hindlimbs we investigated the pattern of spontaneous locomotor behavior along a horizontal runway.

We found that the effects of SNC and treatment with Riluzole on locomotor behavior were reflected by systematic changes in the relationship between the burst durations of EMG muscle activity and the duration of the cycle. The main effects of SNC not previously reported in the literature were: prolongation of EMG burst duration in both EDL muscles resulting in prolongation of co-activation of Sol and EDL muscles and a loss of correlation between the duty factor of the burst of EMG activity in muscles with SNC and that of the control muscle for both the Sol and EDL muscles. Thus, our analysis showed that rats with 2–8 (Sol) and 10–28 (EDL) MUs in the investigated muscles after SNC at birth failed to develop normal muscle control during locomotion. The novel finding of this study was that rats treated with Riluzole developed almost normal control of both Sol and EDL muscles. This was observed for both Sol muscles and control EDL muscles in rats with 5, 6 (Sol) and 15–29 (EDL) MUs rescued after treatment, while rats with 8 (Sol) and 23–26 (EDL) MUs showed almost normal control of both investigated muscles. In contrast, saline treated animals failed to develop normal independent control of Sol muscles even when the numbers of MUs were greater (6–8 and 16–28) or almost the same (8 and 24–28) as in Riluzole treated rats. The subgroup of saline treated rats failed to develop normal control of both Sol and EDL muscles together even when the numbers of survived MUs were almost the same as in Riluzole treated rats. Our results showed that the effectiveness of treatment depended mainly on the number of MUs in the Sol muscle. This result can be explained taking into consideration the fact that normal EDL muscle burst duration is generated with the involvement of sensory information processing producing inhibition of the EDL muscle burst during the stance phase based on the activity of the Sol muscle. Thus, we showed also that the number of motor units, (as shown [5]) was not the only factor in determination of treatment effectiveness. In addition, the results of our present study suggested that prolongation of burst duration of EDL muscle EMG activity was a result of malfunction of neuronal circuits, which reduce bursts of EDL muscle activity, after the onset of activity in the Sol muscle. Taken together, our results support hypothesis that treatment with Riluzole enabled the rats with SNC inflicted at birth to develop almost normal control of both Sol and EDL muscles during locomotion.

Our results are difficult to compare with data in the literature due to differences in the methods of injury and data analysis. However, the locomotor EMG activity of Sol and EDL muscles with SNC inflicted early after birth has been assessed in two studies by Navarrete and Vrbová [23] and Vejsada and colleagues [24]. Furthermore, data from quantitative analysis of muscle activity performed by Sławińska and colleagues [25, 26, 27] has been obtained for muscles with partial denervation. In none of these studies was the activity of both control muscles and muscles with nerve injury recorded simultaneously for the Sol and EDL muscles. Thus, the effect of injury on the relationships between the EMG activity of muscles with SNC and control muscles as well as between flexor-extensor muscles could not be revealed. Moreover, the effect of treatment with Riluzole and the relationship between this effect and the number of rescued MUs was not studied.

The results of the present study concerning the effect of SNC on the burst duration of EMG activity are generally in agreement with those obtained in these previous studies. Prolongation of the activity of the EDL muscle and shortening of the burst duration of Sol muscle activity associated with the abnormal activity of re-innervated tibialis anterior muscles with “extensor like” bursts during the stance phase of step was reported in rats with SNC at 1^st^ day a.b. [23, 24]. Similarly, studies on rats with 13–17 MUs in Sol muscles after partial denervation inflicted at 5 days of age [25, 26, 27] showed the activity of this muscle was shorter by 3 to 36% compared to the control muscle. The observed decrease in the activity duration was consistent with the decrease in the regression slope. In contrast to the activity of Sol muscles, the activity of EDL muscles partially denervated at 3 and 18 days of age was longer by 20–55% as compared to the control muscle consistently with an increase in the slope of regression relating the duration of activity with the duration of cycle.

Full recovery of the performance of hindlimbs after SNC in adult rats found in previous studies, looking at duty factors of hindlimb swing and stance phases [15, 16] is inconsistent with the result of studies performed using analysis of other locomotor indices on adult and newborn rats [20, 21]. The results of this study showed that SNC affected not only the duty factors of muscle EMG activity but also reduced the correlation between these indices below the level found in normal locomotion. This indicates that analysis of the duty factor of the step cycle phase or muscle EMG activity is not sufficient to assess the effect of SNC on muscle control during locomotion and indicated the need for analysis of the relationship between the respective gait indices.

### Mechanism of SNC induced deficits in EDL muscle EMG

Previous studies [23, 24] performed on rats with SNC inflicted at 1^st^ day a.b. showed that the reinnervated flexor muscles (EDL and tibialis anterior, respectively) were activated abnormally with an "extensor-like" burst during the stance phase of the step, while control tibialis anterior muscles were active only during the swing phase. However, the activity of control EDL muscles and co-activation of flexor-extensor muscles has not been previously described.

The activity of extensor and flexor muscles was recorded simultaneously on both sides by Gramsbergen and colleagues [28] and on the injured side by Sabatier and colleagues [29] after transection and repair of sciatic nerve in adult rats. These studies showed that locomotor activity in flexor and extensor muscles was no longer reciprocal but appeared as co-activation. Thus, authors suggested that co-activation of tibialis anterior and soleus muscles was due to “the limited ability for integration of afferent feedback following peripheral nerve injury” [29]. Similarly, it was reported that gastrocnemius and tibialis anterior muscles showed co-activation and often tonic activity of the tibialis anterior muscle on the operated side [28] in addition to a burst of tibialis anterior muscle activity on the unoperated side, which was named “compensational activity”. However, quantitative analysis of the relationship between the EMG activity observed in the investigated muscles has not been performed and so detailed comparison with the results of the current study was not possible.

We propose that deficits in control of EDL muscles induced by SNC were due to the decrease in the number of MUs and also to malfunction of neuronal circuits involved in muscle control induced by the lack of appropriate inhibition in the spinal cord. This was supported by the fact that in contrast to saline treated rats, Riluzole treated rats developed normal activity of both Sol and EDL muscles when the numbers of MUs were the same and developed normal activity of control EDL muscles and of both Sol muscles even when the numbers of MUs were fewer than in saline treated animals. Systematic increases in the duration of EDL muscle activity was found in saline treated rats consistent with an increase in the strength of the relationship between the burst durations of EDL muscle activity and the cycle duration, which was induced by the lack of attenuation of EDL muscle activity during the stance phase, suggesting malfunction of neuronal circuits due to the lack of appropriate inhibition in the spinal cord.

In summary, we can suggest that prolongation of EDL muscle activity in both muscles was a common effect of SNC induced by malfunction of neuronal circuits, which prevented attenuation of EDL muscle activity after the onset of the respective Sol muscle activity. Moreover, malfunction of neuronal circuits involved in the control of EDL muscles with SNC can be caused by the lack of inhibitory feedback from the ipsilateral Sol muscle afferents through the contribution of reciprocal inhibition. There is data indicating that peripheral nerve injury diminishes sensory encoding and afferent synaptic transmission on spinal circuits, which in turn deprive these circuits from inhibitory feedback [2, 17, 38] but these data were obtained from experiments on cat or with the use of nerve dissection. However, data indicating that SNC induced degeneration of muscle receptors [8, 9, 10] is in support of our suggestion that normal sensory encoding was diminished after nerve injury. A similar explanation of the effect SNC on the control of EDL muscles would be possible if EDL muscle activity was reduced during the stance phase, due to the contribution of sensory information from the contralateral limb. This explanation cannot be supported directly by experimental data, however, data obtained by Hayes and colleagues [39] suggested that “presynaptic inhibition binds the sensorimotor states of the two limbs, adjusting sensory inflow to the swing limb based on forces generated by the stance limb”, which supports the suggestion that prolongation of control EDL muscle activity could be associated with a lack of contralateral sensory feedback. However, co-activation of Sol and EDL muscles in our data cannot be considered as a compensatory strategy stabilizing the ankle during the stance phase of limb movement driven by muscles with small number of MUs, because co-activation was observed also in contralateral control limb driven by muscles with a normal numbers of MUs.

### Effectiveness of treatment with Riluzole

Our previous results showed that the number of MUs rescued in the Sol and EDL muscles [5] was not the only determinant of treatment effectiveness. In the present study we demonstrated that rats treated with Riluzole developed almost normal control of EDL muscles due to an action on motoneurons and neuronal circuits, which attenuated the burst of activity of EDL muscle with SNC during the stance phase, which in turn enabled normal activity of control EDL muscle. This effect could occur due to the recovery of reciprocal inhibition from the Sol muscle with SNC. Although experimental data on the neuroprotective effects of Riluzole on neuronal are scarce and were not obtained in rats with SNC inflicted early after birth, a few studies show that treatment with Riluzole affects the circuits involved in muscle control during locomotion. Bergerot and colleagues showed that Riluzole enhanced dendrite formation, which improved locomotor function assessed with the BBB test in rats that had reimplantation of the ventral root after avulsion injury [31]. Studies *in vitro* showed that, in addition to an effect on motoneurons, Riluzole had growth-promoting effects on sensory neurons, by promoting neuritogenesis, neurite branching and enhanced outgrowth both in neonatal and adult cultures [15, 40] promoting sensory afferent regeneration by increasing the production of neurotrophins, which compensated for the loss of trophic support following peripheral nerve injury. Mizuta and also Caumont and colleagues [41, 42] showed that Riluzole stimulates synthesis of nerve growth factor providing some evidence that Riluzole may exert neuroprotective effects on neuronal circuits involved in transmission of sensory information stimulating the production of neurotrophic factors.

In summary: in view of data on the role of afferent information in control of muscle activity during locomotion [43–47] we suggest that treatment with Riluzole enabled our rats with SNC inflicted at the 1^st^ and 2^nd^ day a.b. to develop almost normal control of Sol and EDL muscles not only due to a neuroprotective action on motoneurons but also an action on circuits of afferent feedback involved in muscle control, which protected them against the effect of sciatic nerve crush.

## Supporting Information

S1 Table The phase shift and strength of intralimb and interlimb coordination. The table contains mean (± circular SD) of phase shifts of intralimb (L/Co Sol—L/Co EDL and R/SNC Sol—R/SNC EDL) and interlimb (R/SNC Sol—L/Co Sol and R/SNC EDL—L/Co EDL) coordination and ***r***-values obtained with Polar Plot analysis in individual rats and in groups of intact, saline and Riluzole treated animals. The values of SEM ranged from 0.64 to 1.99%. Abbreviations: L/Co-left/control, R/SNC-right-muscle with SNC, Sol-soleus, EDL-extensor digitorum longus. Abbreviations for statistical significance vs intact rats: *—*p* < 0.001.
(DOC)

S2 Table The duration of cycle. The table contains mean (±SD) of cycle durations established based on the left and right Sol and EDL muscles, on control muscles and muscles with SNC in individual rats and in groups of intact, saline and Riluzole treated animals. The values of SEM ranged from 1.78 to 3.20%. Abbreviations: L/Co-left/control, R/SNC-right/muscle with SNC.
(DOC)

S3 Table The relationship between the burst duration of Sol muscle EMG activity and the duration of cycle. The table contains slopes and intercepts of regression with the values of *p* for significance of intercepts and correlation coefficients *r* in individual intact, saline and Riluzole treated animals. The values of *p* for significance of slopes and correlation coefficients were < 0.001 in all instances. Abbreviations: L/Co-left/control, R/SNC-right/muscle with SNC.
(DOC)

S4 Table The relationship between the burst duration of EDL muscle EMG activity and the duration of cycle. The table contains slopes and intercepts of regression with the values of *p* for their significance as well as correlation coefficients *r* in individual intact, saline and Riluzole treated animals. Abbreviations: L/Co-left/control, R/SNC-right/muscle with SNC.
(DOC)

S5 Table The duration and duty factor of burst of Sol muscle EMG activity. The table contains mean (±SD) cycle duration, burst duration and duty factor in individual rats and in groups of intact, saline and Riluzole treated animals. The values of SEM ranged from 0.71 to 1.67%. Abbreviations: L/Co-left/control, R/SNC-right/muscle with SNC. Abbreviations for statistical significance vs intact rats: *—*p* < 0.011.
(DOC)

S6 Table The duration and duty factor of burst of EDL muscle EMG activity. The table contains mean (±SD) cycle duration, burst duration and duty factor in individual rats and in groups of intact, saline and Riluzole treated animals. The values of SEM ranged from 0.57 to 3.96%. Abbreviations: L/Co-left/control, R/SNC-right/muscle with SNC. Abbreviations for statistical significance vs intact rats: *—*p* < 0.001.
(DOC)

S7 Table The relationship between the duty factor established for right and left hindlimbs (with SNC and control). The table contains correlation coefficients *r* with corresponding values of *p* for significance, for the relationship between the duty factor of burst of EMG activity of right muscle/muscle with SNC and the duty factor of burst of EMG activity of left muscle/control muscle for the Sol and EDL muscles in individual intact rats, saline and Riluzole treated animals. In addition the table contains the values of common correlation coefficients *r*_w_ with the values of *p*_w_ obtained with a test for the heterogeneity of correlation coefficients in the respective groups. Abbreviations: DF-duty factor, Sol-soleus, EDL-extensor digitorum longus.
(DOC)

S8 Table The duration of IS-E and IE-S intervals and the relationship with the burst duration of Sol muscle EMG activity. The table contains mean (±SD) of interval durations, predicted durations, slopes and intercepts of regressions as well as correlation coefficients *r* with the values of *p* obtained in individual rats and in group of intact rats for the left muscles as well as in individual rats and in groups of saline and Riluzole treated animals for the control muscles. The values of SEM ranged from 1.19 to 3.83%. Abbreviations for statistical significance vs intact rats: *—*p* < 0.001.
(DOC)

S9 Table The duration of IS-E and IE-S intervals and the relationship with the burst duration of Sol muscle EMG activity. The table contains mean (±SD) of interval durations, predicted durations, slopes and intercepts of regressions as well as correlation coefficients *r* with the values of *p* obtained in individual rats and in group of intact rats for the right muscles as well as in individual rats and in groups of saline and Riluzole treated animals for muscles with SNC. The values of SEM ranged from 1.19 to 3.83%. Abbreviations for statistical significance vs intact rats: *—*p* < 0.001.
(DOC)
