# Correction: Treatment with Riluzole Restores Normal Control of Soleus and Extensor Digitorum Longus Muscles during Locomotion in Adult Rats after Sciatic Nerve Crush at Birth

**DOI:** 10.1371/journal.pone.0176490

**Published:** 2017-04-20

**Authors:** Wojciech Zmysłowski, Anna M. Cabaj, Urszula Sławińska

The supporting information tables incorrectly include tracked changes that make them illegible. Please view the correct S1–S9 Tables here.

## Supporting information

S1 Table The phase shift and strength of intralimb and interlimb coordination. The table contains mean (± circular SD) of phase shifts of intralimb (L/Co Sol—L/Co EDL and R/SNC Sol—R/SNC EDL) and interlimb (R/SNC Sol—L/Co Sol and R/SNC EDL—L/Co EDL) coordination and ***r***-values obtained with Polar Plot analysis in individual rats and in groups of intact, saline and Riluzole treated animals. The values of SEM ranged from 0.64 to 1.99%. Abbreviations: L/Co-left/control, R/SNC-right-muscle with SNC, Sol-soleus, EDL-extensor digitorum longus. Abbreviations for statistical significance vs intact rats: *—*p* < 0.001.
(DOC)

S2 Table The duration of cycle. The table contains mean (±SD) of cycle durations established based on the left and right Sol and EDL muscles, on control muscles and muscles with SNC in individual rats and in groups of intact, saline and Riluzole treated animals. The values of SEM ranged from 1.78 to 3.20%. Abbreviations: L/Co-left/control, R/SNC-right/muscle with SNC.
(DOC)

S3 Table The relationship between the burst duration of Sol muscle EMG activity and the duration of cycle. The table contains slopes and intercepts of regression with the values of *p* for significance of intercepts and correlation coefficients *r* in individual intact, saline and Riluzole treated animals. The values of *p* or significance of slopes and correlation coefficients were < 0.001 in all instances. Abbreviations: L/Co-left/control, R/SNC-right/muscle with SNC.
(DOC)

S4 Table The relationship between the burst duration of EDL muscle EMG activity and the duration of cycle. The table contains slopes and intercepts of regression with the values of *p* for their significance as well as correlation coefficients *r* in individual intact, saline and Riluzole treated animals. Abbreviations: L/Co-left/control, R/SNC-right/muscle with SNC.
(DOC)

S5 Table The duration and duty factor of burst of Sol muscle EMG activity. The table contains mean (±SD) cycle duration, burst duration and duty factor in individual rats and in groups of intact, saline and Riluzole treated animals. The values of SEM ranged from 0.71 to 1.67%. Abbreviations: L/Co-left/control, R/SNC-right/muscle with SNC. Abbreviations for statistical significance vs intact rats: *—*p* < 0.011.
(DOC)

S6 Table The duration and duty factor of burst of EDL muscle EMG activity. The table contains mean (±SD) cycle duration, burst duration and duty factor in individual rats and in groups of intact, saline and Riluzole treated animals. The values of SEM ranged from 0.57 to 3.96%. Abbreviations: L/Co-left/control, R/SNC-right/muscle with SNC. Abbreviations for statistical significance vs intact rats: *—*p* < 0.001.
(DOC)

S7 Table The relationship between the duty factor established for right and left hindlimbs (with SNC and control). The table contains correlation coefficients *r* with corresponding values of *p* for significance, for the relationship between the duty factor of burst of EMG activity of right muscle/muscle with SNC and the duty factor of burst of EMG activity of left muscle/control muscle for the Sol and EDL muscles in individual intact rats, saline and Riluzole treated animals. In addition the table contains the values of common correlation coefficients *r*_w_ with the values of *p*_w_ obtained with a test for the heterogeneity of correlation coefficients in the respective groups. Abbreviations: DF-duty factor, Sol-soleus, EDL-extensor digitorum longus.
(DOC)

S8 Table The duration of IS-E and IE-S intervals and the relationship with the burst duration of Sol muscle EMG activity. The table contains mean (±SD) of interval durations, predicted durations, slopes and intercepts of regressions as well as correlation coefficients *r* with the values of *p* obtained in individual rats and in group of intact rats for the left muscles as well as in individual rats and in groups of saline and Riluzole treated animals for the control muscles. The values of SEM ranged from 1.19 to 3.83%. Abbreviations for statistical significance vs intact rats: *—*p* < 0.001.
(DOC)

S9 Table The duration of IS-E and IE-S intervals and the relationship with the burst duration of Sol muscle EMG activity. The table contains mean (±SD) of interval durations, predicted durations, slopes and intercepts of regressions as well as correlation coefficients *r* with the values of *p* obtained in individual rats and in group of intact rats for the right muscles as well as in individual rats and in groups of saline and Riluzole treated animals for muscles with SNC. The values of SEM ranged from 1.19 to 3.83%. Abbreviations for statistical significance vs intact rats: *—*p* < 0.001.
(DOC)
