# Supplementary material for: Treatment with Riluzole Restores Normal Control of Soleus and Extensor Digitorum Longus Muscles during Locomotion in Adult Rats after Sciatic Nerve Crush at Birth
Source: PLoS One. 2017 Jan 17;12(1):e0170235. doi: 10.1371/journal.pone.0170235 (PMC5240973; doi:10.1371/journal.pone.0170235)
Supplement: S1 Table — The table contains mean (± circular SD) of phase shifts of intralimb (L/Co Sol—L/Co EDL and R/SNC Sol—R/SNC EDL) and interlimb (R/SNC Sol—L/Co Sol and R/SNC EDL—L/Co EDL) coordination and r-values obtained with Polar Plot analysis in individual rats and in groups of intact, saline and Riluzole treated animals. The values of SEM ranged from 0.64 to 1.99%. Abbreviations: L/Co-left/control, R/SNC-right-muscle with SNC, Sol-soleus, EDL-extensor digitorum longus. Abbreviations for statistical significance vs intact rats: *—p < 0.001. (DOC) [file pone.0170235.s001.doc]

**S1 Table. The phase shift and strength of intralimb and interlimb coordination.**

|  |  | Intralimb  coordi-  nation |  |  |  | Interlimb  coordi-  nation |  |  |  |
| --- | --- | --- | --- | --- | --- | --- | --- | --- | --- |
| Group | Rat | Phase  shift L/Co  muscles | ***r*** | Phase  shift R/SNC  muscles | ***r*** | Phase  shift  Sol/Sol  muscles | ***r*** | Phase  shift EDL/EDL  muscles | ***r*** |
|  |  | [deg] |  | [deg] |  | [deg] |  | [deg] |  |
|  |  |  |  |  |  |  |  |  |  |
|  | IN1 | 112±14 | 0.97 | 101±12 | 0.98 | 185±8 | 0.99 | 191±28 | 0.89 |
| IN | IN2 | 114±18 | 0.95 | 106±18 | 0.95 | 178±12 | 0.98 | 165±12 | 0.98 |
|  | IN3 | 115±16 | 0.96 | 102±14 | 0.97 | 170±8 | 0.99 | 169±26 | 0.93 |
|  | Group | 113±20 | 0.94 | 103±16 | 0.96 | 178±14 | 0.97 | 175±26 | 0.90 |
|  |  |  |  |  |  |  |  |  |  |
|  | NB4 | 113±23 | 0.92 | 107±23 | 0.92 | 216±14 | 0.97 | 208±39 | 0.79 |
|  | NB5 | 99±26 | 0.90 | 90±20 | 0.94 | 199±20 | 0.94 | 213±38 | 0.80 |
| 1S | NB2 | 100±26 | 0.90 | 94±23 | 0.92 | 184±26 | 0.90 | 195±36 | 0.82 |
|  | NB6 | 102±29 | 0.88 | 117±28 | 0.89 | 204±20 | 0.94 | 215±34 | 0.84 |
|  | Group | 103±29 | 0.88 | 102±28 | 0.89 | 201±28* | 0.89 | 209±39* | 0.79 |
|  |  |  |  |  |  |  |  |  |  |
|  | NA4 | 93±20 | 0.96 | 99±18 | 0.95 | 178±23 | 0.92 | 176±37 | 0.81 |
|  | NA5 | 82±34 | 0.84 | 93±23 | 0.92 | 198±25 | 0.91 | 203±39 | 0.79 |
| 2S | NA7 | 104±12 | 0.98 | 89±12 | 0.98 | 196±25 | 0.91 | 222±34 | 0.84 |
|  | NA6 | 81±22 | 0.93 | 94±18 | 0.95 | 187±14 | 0.97 | 176±38 | 0.80 |
|  | KB6 | 62±8 | 0.99 | 106±20 | 0.94 | 189±26 | 0.90 | 165±34 | 0.84 |
|  | Group | 86±26* | 0.90 | 96±22* | 0.93 | 190±26 | 0.90 | 189±37 | 0.81 |
|  |  |  |  |  |  |  |  |  |  |
|  | RA1 | 88±18 | 0.95 | 148±20 | 0.94 | 217±22 | 0.93 | 171±29 | 0.88 |
|  | RA4 | 84±16 | 0.96 | 121±16 | 0.96 | 229±28 | 0.89 | 192±33 | 0.85 |
| RG1 | RA6 | 96±14 | 0.97 | 108±29 | 0.88 | 180±16 | 0.96 | 176±20 | 0.94 |
|  | RB4 | 90±22 | 0.93 | 90±23 | 0.92 | 185±14 | 0.97 | 197±20 | 0.94 |
|  | RB5 | 121±35 | 0.83 | 107±12 | 0.98 | 203±23* | 0.92 | 207±33 | 0.85 |
|  | Group | 96±26 | 0.90 | 119±31* | 0.86 | 203±29 | 0.88 | 190±34 | 0.84 |
|  |  |  |  |  |  |  |  |  |  |
|  | RB6 | 100±22 | 0.93 | 104±20 | 0.94 | 190±8 | 0.99 | 186±28 | 0.89 |
|  | RB7 | 112±29 | 0.88 | 110±26 | 0.90 | 187±22 | 0.93 | 196±37 | 0.81 |
| RG2 | RA5 | 96±20 | 0.94 | 124±25 | 0.91 | 172±33 | 0.85 | 153±38 | 0.80 |
|  | RA11 | 107±12 | 0.98 | 107±23 | 0.92 | 188±29 | 0.88 | 198±26 | 0.82 |
|  | Group | 104±25 | 0.91 | 110±26 | 0.90 | 184±26 | 0.90 | 181±37 | 0.81 |
|  |  |  |  |  |  |  |  |  |  |
|  |  |  |  |  |  |  |  |  |  |

The table contains mean (± circular SD) of phase shifts of intralimb (L/Co Sol - L/Co EDL and R/SNC Sol - R/SNC EDL) and interlimb (R/SNC Sol - L/Co Sol and R/SNC EDL - L/Co EDL) coordination and ***r***-values obtained with Polar Plot analysis in individual rats and in groups of intact, saline and Riluzole treated animals. The values of SEM ranged from 0.64 to 1.99%. Abbreviations: L/Co-left/control, R/SNC-right/muscle with SNC, Sol-soleus, EDL-extensor digitorum longus. Abbreviations for statistical significance vs intact rats: * - *p* < 0.001.
