# Supplementary material for: Treatment with Riluzole Restores Normal Control of Soleus and Extensor Digitorum Longus Muscles during Locomotion in Adult Rats after Sciatic Nerve Crush at Birth
Source: PLoS One. 2017 Jan 17;12(1):e0170235. doi: 10.1371/journal.pone.0170235 (PMC5240973; doi:10.1371/journal.pone.0170235)
Supplement: S2 Table — The table contains mean (±SD) of cycle durations established based on the left and right Sol and EDL muscles, on control muscles and muscles with SNC in individual rats and in groups of intact, saline and Riluzole treated animals. The values of SEM ranged from 1.78 to 3.20%. Abbreviations: L/Co-left/control, R/SNC-right/muscle with SNC. (DOC) [file pone.0170235.s002.doc]

**S2 Table. The duration of cycle.**

|  |  | Sol |  | EDL |  |
| --- | --- | --- | --- | --- | --- |
| Group | Rat | L/Co | R/SNC | L/Co | R/SNC |
|  |  | [ms] | [ms] | [ms] | [ms] |
|  |  |  |  |  |  |
|  | IN1 | 333±73 | 337±76 | 332±77 | 331±71 |
| IN | IN2 | 320±73 | 316±72 | 328±84 | 322±76 |
|  | IN3 | 311±68 | 308±60 | 308±57 | 314±62 |
|  | Group | 322±71 | 320±70 | 321±75 | 322±73 |
|  |  |  |  |  |  |
|  | NB4 | 394±128 | 396±131 | 388±131 | 383±127 |
|  | NB5 | 458±105 | 462±111 | 464±100 | 454±105 |
| 1S | NB2 | 299±119 | 301±119 | 300±127 | 292±119 |
|  | NB6 | 320±130 | 315±126 | 328±135 | 312±123 |
|  | Group | 367±138 | 368±141 | 369±142 | 362±138 |
|  |  |  |  |  |  |
|  | NA4 | 333±72 | 337±73 | 343±77 | 342±75 |
|  | NA5 | 374±133 | 363±125 | 392±129 | 386±103 |
| 2S | NA7 | 349±82 | 349±80 | 341±72 | 339±76 |
|  | NA6 | 331±88 | 326±80 | 316±70 | 318±76 |
|  | KB6 | 449±100 | 449±92 | 457±100 | 453±92 |
|  | Group | 367±103 | 364±102 | 360±100 | 358±100 |
|  |  |  |  |  |  |
|  | RA1 | 259±73 | 251±59 | 257±79 | 255±62 |
|  | RA4 | 297±88 | 293±80 | 285±87 | 280±78 |
| RG1 | RA6 | 311±85 | 305±77 | 308±68 | 308±97 |
|  | RB4 | 358±84 | 348±72 | 348±68 | 339±62 |
|  | RB5 | 324±112 | 314±100 | 327±106 | 320±98 |
|  | Group | 311±92 | 302±83 | 303±92 | 299±86 |
|  |  |  |  |  |  |
|  | RB6 | 343±106 | 329±93 | 351±114 | 334±110 |
|  | RB7 | 336±116 | 331±107 | 338±126 | 329±109 |
| RG2 | RA5 | 293±92 | 287±85 | 295±97 | 289±91 |
|  | RA11 | 346±93 | 342±89 | 322±83 | 318±74 |
|  | Group | 323±104 | 322±96 | 329±110 | 319±97 |
|  |  |  |  |  |  |

The table contains mean (±SD) of cycle durations established based on the left and right Sol and EDL muscles, on control muscles and muscles with SNC in individual rats and in groups of intact, saline and Riluzole treated animals. The values of SEM ranged from 1.78 to 3.20%. Abbreviations: L/Co-left/control, R/SNC-right/muscle with SNC.
