# Supplementary material for: Treatment with Riluzole Restores Normal Control of Soleus and Extensor Digitorum Longus Muscles during Locomotion in Adult Rats after Sciatic Nerve Crush at Birth
Source: PLoS One. 2017 Jan 17;12(1):e0170235. doi: 10.1371/journal.pone.0170235 (PMC5240973; doi:10.1371/journal.pone.0170235)
Supplement: S3 Table — The table contains slopes and intercepts of regression with the values of p for significance of intercepts and correlation coefficients r in individual intact, saline and Riluzole treated animals. The values of p for significance of slopes and correlation coefficients were < 0.001 in all instances. Abbreviations: L/Co-left/control, R/SNC-right/muscle with SNC. (DOC) [file pone.0170235.s003.doc]

**S3 Table. The relationship between the burst duration of Sol muscle EMG activity and the duration of cycle.**

|  |  | L/Co |  |  |  | R/SNC |  |  |  |
| --- | --- | --- | --- | --- | --- | --- | --- | --- | --- |
| Group | Rat | Slope | Intercept | *p* intercept | *r* | Slope | Intercept | *p* intercept | *r* |
|  |  |  | [ms] |  |  |  | [ms] |  |  |
|  |  |  |  |  |  |  |  |  |  |
|  | IN1 | 0.85 | -91 | <0.001 | 0.959 | 0.84 | -76 | <0.001 | 0.936 |
| IN | IN2 | 0.82 | -88 | <0.001 | 0.928 | 0.89 | -91 | <0.001 | 0.960 |
|  | IN3 | 0.85 | -89 | <0.001 | 0.956 | 0.85 | -76 | <0.001 | 0.927 |
|  |  |  |  |  |  |  |  |  |  |
|  | NB4 | 0.79 | -73 | <0.001 | 0.959 | 0.56 | -3 | 0.878 | 0.927 |
|  | NB5 | 0.74 | -84 | <0.001 | 0.843 | 0.38 | -12 | 0.672 | 0.648 |
| 1S | NB2 | 0.74 | -57 | <0.001 | 0.941 | 0.61 | -13 | 0.161 | 0.995 |
|  | NB6 | 0.76 | -49 | <0.001 | 0.980 | 0.57 | -16 | 0.277 | 0.859 |
|  |  |  |  |  |  |  |  |  |  |
|  | NA4 | 0.86 | -79 | <0.001 | 0.980 | 0.62 | -14 | 0.126 | 0.959 |
|  | NA5 | 0.99 | -129 | <0.001 | 0.958 | 0.53 | -11 | 0.241 | 0.943 |
| 2S | NA7 | 0.85 | -93 | <0.001 | 0.980 | 0.69 | -15 | 0.325 | 0.911 |
|  | NA6 | 0.83 | -49 | <0.001 | 0.956 | 0.76 | -20 | 0.131 | 0.959 |
|  | KB6 | 0.88 | -93 | <0.001 | 0.938 | 0.56 | -2 | 0.972 | 0.768 |
|  |  |  |  |  |  |  |  |  |  |
|  | RA1 | 0.95 | -82 | <0.001 | 0.972 | 0.74 | -64 | <0.001 | 0.959 |
|  | RA4 | 0.84 | -57 | <0.001 | 0.957 | 0.79 | -46 | <0.001 | 0.919 |
| RG1 | RA6 | 0.83 | -86 | <0.001 | 0.964 | 0.80 | -70 | <0.001 | 0.885 |
|  | RB4 | 0.74 | -65 | <0.001 | 0.897 | 0.73 | -64 | <0.001 | 0.959 |
|  | RB5 | 0.76 | -54 | <0.001 | 0.962 | 0.76 | -50 | <0.001 | 0.949 |
|  |  |  |  |  |  |  |  |  |  |
|  | RB6 | 0.73 | -57 | <0.001 | 0.947 | 0.78 | -55 | <0.001 | 0.960 |
|  | RB7 | 0.74 | -59 | <0.001 | 0.964 | 0.70 | -61 | <0.001 | 0.917 |
| RG2 | RA5 | 0.86 | -70 | <0.001 | 0.952 | 0.76 | -55 | <0.001 | 0.938 |
|  | RA11 | 0.73 | -66 | <0.001 | 0.967 | 0.63 | -51 | <0.001 | 0.902 |
|  |  |  |  |  |  |  |  |  |  |

The table contains slopes and intercepts of regression with the values of *p* for significance of intercepts as well as correlation coefficients *r* in individual intact, saline and Riluzole treated animals. The values of *p* for significance of slopes and correlation coefficients were < 0.001 in all instances. Abbreviations: L/Co-left/control, R/SNC-right/muscle with SNC.
