# Supplementary material for: Treatment with Riluzole Restores Normal Control of Soleus and Extensor Digitorum Longus Muscles during Locomotion in Adult Rats after Sciatic Nerve Crush at Birth
Source: PLoS One. 2017 Jan 17;12(1):e0170235. doi: 10.1371/journal.pone.0170235 (PMC5240973; doi:10.1371/journal.pone.0170235)
Supplement: S4 Table — The table contains slopes and intercepts of regression with the values of p for their significance as well as correlation coefficients r in individual intact, saline and Riluzole treated animals. Abbreviations: L/Co-left/control, R/SNC-right/muscle with SNC. (DOC) [file pone.0170235.s004.doc]

**S4 Table. The relationship between the burst duration of EDL muscle EMG activity and the duration of cycle.**

|  |  | L/Co |  |  |  |  | R/SNC |  |  |  |  |
| --- | --- | --- | --- | --- | --- | --- | --- | --- | --- | --- | --- |
| Group | Rat | Slope | Inter-  cept | *p*  inter-  cept | *r* | *p*  slope | Slope | Inter-  cept | *p*  inter-  cept | *r* | *p*  slope |
|  |  |  | [ms] |  |  |  |  | [ms] |  |  |  |
|  |  |  |  |  |  |  |  |  |  |  |  |
|  | IN1 | 0.06 | 158 | <0.001 | 0.138 | 0.339 | 0.07 | 152 | <0.001 | 0.187 | 0.096 |
| IN | IN2 | 0.21 | 99 | <0.001 | 0.445 | 0.001 | 0.06 | 138 | <0.001 | 0.200 | 0.081 |
|  | IN3 | 0.15 | 109 | <0.001 | 0.416 | 0.003 | 0.11 | 126 | <0.001 | 0.302 | 0.033 |
|  |  |  |  |  |  |  |  |  |  |  |  |
|  | NB4 | 0.92 | -24 | 0.069 | 0.964 | <0.001 | 0.52 | 76 | 0.014 | 0.840 | <0.001 |
|  | NB5 | 0.59 | 63 | 0.034 | 0.792 | <0.001 | 0.74 | -8 | 0.721 | 0.887 | <0.001 |
| 1S | NB2 | 0.84 | -2 | 0.880 | 0.917 | <0.001 | 0.77 | 10 | 0.251 | 0.964 | <0.001 |
|  | NB6 | 0.77 | 1 | 0.928 | 0.922 | <0.001 | 0.73 | 34 | 0.008 | 0.933 | <0.001 |
|  |  |  |  |  |  |  |  |  |  |  |  |
|  | NA4 | 0.89 | -4 | 0.741 | 0.964 | <0.001 | 0.81 | 3 | 0.875 | 0.927 | <0.001 |
|  | NA5 | 0.87 | -6 | 0.691 | 0.954 | <0.001 | 0.71 | 1 | 0.936 | 0.917 | <0.001 |
| 2S | NA7 | 0.75 | 39 | 0.089 | 0.860 | <0.001 | 0.64 | 61 | 0.015 | 0.851 | <0.001 |
|  | NA6 | 0.86 | 15 | 0.593 | 0.954 | <0.001 | 0.81 | 25 | 0.030 | 0.949 | <0.001 |
|  | KB6 | 0.87 | -15 | 0.206 | 0.866 | <0.001 | 0.73 | 73 | 0.092 | 0.837 | <0.001 |
|  |  |  |  |  |  |  |  |  |  |  |  |
|  | RA1 | 0.03 | 123 | <0.001 | 0.130 | 0.368 | 0.71 | 33 | 0.026 | 0.873 | <0.001 |
|  | RA4 | 0.07 | 96 | <0.001 | 0.184 | 0.200 | 0.66 | 36 | 0.024 | 0.859 | <0.001 |
| RG1 | RA6 | 0.02 | 110 | 0.002 | 0.045 | 0.756 | 0.46 | 88 | <0.001 | 0.823 | <0.001 |
|  | RB4 | 0.04 | 133 | <0.001 | 0.127 | 0.379 | 0.45 | 97 | <0.001 | 0.711 | <0.001 |
|  | RB5^1^ | 0.84 | 5 | 0.810 | 0.898 | <0.001 | 0.86 | 7 | 0.532 | 0.956 | <0.001 |
|  |  |  |  |  |  |  |  |  |  |  |  |
|  | RB6 | 0.17 | 101 | <0.001 | 0.332 | 0.009 | 0.02 | 111 | <0.001 | 0.192 | 0.181 |
|  | RB7 | 0.23 | 75 | <0.001 | 0.490 | 0.001 | 0.26 | 87 | <0.001 | 0.749 | <0.001 |
| RG2 | RA5 | 0.18 | 100 | <0.001 | 0.650 | <0.001 | 0.14 | 85 | <0.001 | 0.574 | <0.001 |
|  | RA11 | 0.11 | 141 | <0.001 | 0.323 | 0.011 | 0.09 | 139 | <0.001 | 0.241 | 0.045 |
|  |  |  |  |  |  |  |  |  |  |  |  |

The table contains slopes and intercepts of regression with the values of *p* for their significance as well as correlation coefficients *r* in individual intact, saline and Riluzole treated animals. Abbreviations: L/Co-left/control, R/SNC-right/muscle with SNC.

^1^-denotes data for rat RB5 with abnormal parameters of regression for control muscle.
