# Supplementary material for: Treatment with Riluzole Restores Normal Control of Soleus and Extensor Digitorum Longus Muscles during Locomotion in Adult Rats after Sciatic Nerve Crush at Birth
Source: PLoS One. 2017 Jan 17;12(1):e0170235. doi: 10.1371/journal.pone.0170235 (PMC5240973; doi:10.1371/journal.pone.0170235)
Supplement: S5 Table — The table contains mean (±SD) cycle duration, burst duration and duty factor in individual rats and in groups of intact, saline and Riluzole treated animals. The values of SEM ranged from 0.71 to 1.67%. Abbreviations: L/Co-left/control, R/SNC-right/muscle with SNC. Abbreviations for statistical significance vs intact rats: *—p < 0.011. (DOC) [file pone.0170235.s005.doc]

**S5 Table. The duration and duty factor of burst of Sol muscle EMG activity.**

|  |  | Cycle duration |  | Burst duration |  | Duty factor |  |
| --- | --- | --- | --- | --- | --- | --- | --- |
| Group | Rat | L/Co | R/SNC | L/Co | R/SNC | L/Co | R/SNC |
|  |  | [ms] | [ms] | [ms] | [ms] |  |  |
|  |  |  |  |  |  |  |  |
|  | IN1 | 333±73 | 337±76 | 191±64 | 208±66 | 0.56±0.08 | 0.58±0.08 |
| IN | IN2 | 320±73 | 316±72 | 174±62 | 194±76 | 0.53±0.07 | 0.57±0.09 |
|  | IN3 | 311±68 | 308±60 | 176±60 | 186±55 | 0.56±0.07 | 0.57±0.08 |
|  | Group | 322±71 | 320±70 | 181±62 | 196±66 | 0.55±0.07 | 0.57±0.07 |
|  |  |  |  |  |  |  |  |
|  | NB4 | 394±128 | 396±131 | 240±105 | 216±75 | 0.59±0.09 | 0.55±0.05 |
|  | NB5 | 458±105 | 462±111 | 253±93 | 162±63 | 0.54±0.10 | 0.36±0.09 |
| 1S | NB2 | 299±119 | 301±119 | 167±92 | 174±83 | 0.53±0.10 | 0.57±0.07 |
|  | NB6 | 320±130 | 315±126 | 196±100 | 163±83 | 0.58±0.08 | 0.51±0.07 |
|  | Group | 367±138 | 368±141 | 213±104* | 180±80 | 0.59±0.10 | 0.50±0.11* |
|  |  |  |  |  |  |  |  |
|  | NA4 | 333±72 | 337±73 | 211±63 | 195±50 | 0.62±0.06 | 0.58±0.04 |
|  | NA5 | 374±133 | 363±125 | 245±134 | 189±77 | 0.65±0.12 | 0.50±0.06 |
| 2S | NA7 | 349±82 | 349±80 | 204±70 | 224±58 | 0.58±0.06 | 0.65±0.06 |
|  | NA6 | 331±88 | 326±80 | 227±79 | 220±59 | 0.69±0.07 | 0.69±0.05 |
|  | KB6 | 449±100 | 449±92 | 295±91 | 247±67 | 0.65±0.07 | 0.56±0.09 |
|  | Group | 367±103 | 364±102 | 236±95* | 215±65 | 0.64±0.09* | 0.61±0.09* |
|  |  |  |  |  |  |  |  |
|  | RA1 | 259±73 | 251±59 | 164±72 | 125±46 | 0.61±0.09 | 0.49±0.07 |
|  | RA4 | 297±88 | 293±80 | 161±77 | 175±69 | 0.66±0.08 | 0.60±0.09 |
| RG1 | RA6 | 311±85 | 305±77 | 172±74 | 174±68 | 0.53±0.08 | 0.56±0.08 |
|  | RB4 | 358±84 | 348±72 | 193±51 | 183±43 | 0.53±0.14 | 0.55±0.08 |
|  | RB5 | 324±112 | 314±100 | 193±89 | 185±81 | 0.57±0.08 | 0.59±0.09 |
|  | Group | 311±92 | 302±83 | 176±73 | 167±63* | 0.56±0.10 | 0.55±0.09 |
|  |  |  |  |  |  |  |  |
|  | RB6 | 343±106 | 329±93 | 194±82 | 200±71 | 0.55±0.08 | 0.61±0.06 |
|  | RB7 | 336±116 | 331±107 | 188±86 | 167±75 | 0.55±0.07 | 0.50±0.08 |
| RG2 | RA5 | 293±92 | 287±85 | 185±85 | 163±66 | 0.60±0.10 | 0.57±0.07 |
|  | RA11 | 346±93 | 342±89 | 189±70 | 166±62 | 0.53±0.07 | 0.48±0.09 |
|  | Group | 323±104 | 322±96 | 189±81 | 175±70 | 0.56±0.08 | 0.55±0.09 |
|  |  |  |  |  |  |  |  |

The table contains mean (±SD) cycle duration, burst duration and duty factor in individual rats and in groups of intact, saline and Riluzole treated animals. The values of SEM ranged from 0.71 to 1.67%. Abbreviations: L/Co-left/control, R/SNC-right/muscle with SNC. Abbreviations for statistical significance vs intact rats: * - *p* < 0.011.
