# Supplementary material for: Treatment with Riluzole Restores Normal Control of Soleus and Extensor Digitorum Longus Muscles during Locomotion in Adult Rats after Sciatic Nerve Crush at Birth
Source: PLoS One. 2017 Jan 17;12(1):e0170235. doi: 10.1371/journal.pone.0170235 (PMC5240973; doi:10.1371/journal.pone.0170235)
Supplement: S6 Table — The table contains mean (±SD) cycle duration, burst duration and duty factor in individual rats and in groups of intact, saline and Riluzole treated animals. The values of SEM ranged from 0.57 to 3.96%. Abbreviations: L/Co-left/control, R/SNC-right/muscle with SNC. Abbreviations for statistical significance vs intact rats: *—p < 0.001. (DOC) [file pone.0170235.s006.doc]

**S6 Table.** **The duration and duty factor of burst of EDL muscle EMG activity.**

|  |  | Cycle duration |  | Burst duration |  | Duty factor |  |
| --- | --- | --- | --- | --- | --- | --- | --- |
| Group | Rat | L/Co | R/SNC | L/Co | R/SNC | L/Co | R/SNC |
|  |  | [ms] | [ms] | [ms] | [ms] |  |  |
|  |  |  |  |  |  |  |  |
|  | IN1 | 332±77 | 331±71 | 178±33 | 177±33 | 0.53±0.14 | 0.52±0.11 |
| IN | IN2 | 328±84 | 322±76 | 157±28 | 160±26 | 0.49±0.10 | 0.51±0.11 |
|  | IN3 | 308±57 | 314±62 | 160±32 | 159±28 | 0.51±0.11 | 0.52±0.12 |
|  | Group | 321±75 | 322±73 | 165±32 | 165±30 | 0.51±0.12 | 0.52±0.11 |
|  |  |  |  |  |  |  |  |
|  | NB4 | 388±131 | 383±127 | 333±123 | 307±83 | 0.84±0.08 | 0.78±0.09 |
|  | NB5 | 464±100 | 454±105 | 317±75 | 337±91 | 0.70±0.10 | 0.73±0.07 |
| 1S | NB2 | 300±127 | 292±119 | 250±109 | 238±95 | 0.83±0.11 | 0.80±0.06 |
|  | NB6 | 328±135 | 312±123 | 249±109 | 274±98 | 0.78±0.10 | 0.87±0.06 |
|  | Group | 369±142 | 362±138 | 287±112* | 289±98* | 0.78±0.11* | 0.79±0.09* |
|  |  |  |  |  | , |  |  |
|  | NA4 | 343±77 | 342±75 | 301±68 | 275±55 | 0.88±0.05 | 0.81±0.07 |
|  | NA5 | 392±129 | 386±103 | 330±122 | 256±97 | 0.86±0.07 | 0.71±0.10 |
| 2S | NA7 | 341±72 | 339±76 | 309±70 | 292±59 | 0.86±0.06 | 0.83±0.08 |
|  | NA6 | 316±70 | 318±76 | 309±74 | 296±64 | 0.91±0.04 | 0.90±0.04 |
|  | KB6 | 457±100 | 453±92 | 374±91 | 409±81 | 0.82±0.09 | 0.91±0.06 |
|  | Group | 360±100 | 358±100 | 317±93* | 303±87* | 0.87±0.07* | 0.82±0.11* |
|  |  |  |  |  |  |  |  |
|  | RA1 | 257±79 | 255±62 | 128±19 | 210±46 | 0.49±0.13 | 0.83±0.05 |
|  | RA4 | 285±87 | 280±78 | 129±29 | 225±56 | 0.44±0.13 | 0.78±0.07 |
| RG1 | RA6 | 308±68 | 308±97 | 128±47 | 231±45 | 0.40±0.13 | 0.75±0.10 |
|  | RB4 | 348±68 | 339±62 | 154±32 | 244±40 | 0.42±0.11 | 0.70±0.10 |
|  | RB5^1^ | 327±106 | 320±98 | 282±107 | 280±92 | 0.87±0.14 | 0.88±0.06 |
|  | Group | 303±92 | 299±86 | 133±88* | 238±64* | 0.43±0.22 | 0.79±0.10* |
|  |  |  |  |  |  |  |  |
|  | RB6 | 351±114 | 334±110 | 164±61 | 147±29 | 0.49±0.14 | 0.45±0.10 |
|  | RB7 | 338±126 | 329±109 | 152±44 | 173±36 | 0.47±0.11 | 0.52±0.09 |
| RG2 | RA5 | 295±97 | 289±91 | 153±31 | 125±21 | 0.55±0.10 | 0.43±0.10 |
|  | RA11 | 322±83 | 318±74 | 177±30 | 171±34 | 0.51±0.11 | 0.50±0.13 |
|  | Group | 329±110 | 319±97 | 161±45 | 154±36 | 0.50±0.12 | 0.48±0.11 |
|  |  |  |  |  |  |  |  |

The table contains mean (±SD) cycle duration, burst duration and duty factor in individual rats and in groups of intact, saline and Riluzole treated animals. The values of SEM ranged from 0.57 to 3.96%. Abbreviations: L/Co-left/control, R/SNC-right/muscle with SNC. Abbreviations for statistical significance vs intact rats: * - *p* < 0.001.

^1^-denotes data for rat RB5 with abnormal duty factor of control EDL muscle EMG activity.
