# Supplementary material for: Treatment with Riluzole Restores Normal Control of Soleus and Extensor Digitorum Longus Muscles during Locomotion in Adult Rats after Sciatic Nerve Crush at Birth
Source: PLoS One. 2017 Jan 17;12(1):e0170235. doi: 10.1371/journal.pone.0170235 (PMC5240973; doi:10.1371/journal.pone.0170235)
Supplement: S7 Table — The table contains correlation coefficients r with corresponding values of p for significance, for the relationship between the duty factor of burst of EMG activity of right muscle/muscle with SNC and the duty factor of burst of EMG activity of left muscle/control muscle for the Sol and EDL muscles in individual intact rats, saline and Riluzole treated animals. In addition the table contains the values of common correlation coefficients rw with the values of pw obtained with a test for the heterogeneity of correlation coefficients in the respective groups. Abbreviations: DF-duty factor, Sol-soleus, EDL-extensor digitorum longus. (DOC) [file pone.0170235.s007.doc]

**S7 Table. The relationship between the duty factor established for right and left hindlimb (with SNC and control).**

|  |  | DF Sol  vs  DF Sol |  | DF EDL  vs  DF EDL |  |
| --- | --- | --- | --- | --- | --- |
| Group | Rat | *r/r*_w_ | *p/p*_w_ | *r/r*_w_ | *p/p*_w_ |
|  |  |  |  |  |  |
|  | IN1 | 0.699 | <0.001 | 0.534 | <0.001 |
| IN | IN2 | 0.558 | <0.001 | 0.690 | <0.001 |
|  | IN3 | 0.597 | <0.001 | 0.640 | <0.001 |
|  | Group | 0.625 | 0.527 | 0.640 | 0.463 |
|  |  |  |  |  |  |
|  | NB4 | 0.084 | 0.560 | 0.061 | 0.672 |
|  | NB5 | 0.039 | 0.788 | 0.099 | 0.492 |
| 1S | NB2 | 0.092 | 0.524 | -0.110 | 0.446 |
|  | NB6 | 0.111 | 0.442 | 0.010 | 0.944 |
|  | Group | 0.092 | 0.988 | 0.015 | 0.760 |
|  |  |  |  |  |  |
|  | NA4 | 0.109 | 0.450 | -0.033 | 0.820 |
|  | NA5 | 0.117 | 0.418 | -0.072 | 0.618 |
| 2S | NA7 | 0.091 | 0.528 | -0.054 | 0.708 |
|  | NA6 | 0.084 | 0.560 | -0.076 | 0.598 |
|  | KB6 | -0.109 | 0.568 | -0.077 | 0.594 |
|  | Group | 0.059 | 0.845 | -0.062 | 0.999 |
|  |  |  |  |  |  |
|  | RA1 | 0.636 | <0.001 | 0.010 | 0.944 |
|  | RA4 | 0.493 | <0.001 | 0.148 | 0.304 |
| RG1 | RA6 | 0.553 | <0.001 | 0.070 | 0.608 |
|  | RB4 | 0.424 | 0.001 | -0.118 | 0.414 |
|  | RB5 | 0.469 | <0.001 | 0.143 | 0.320 |
|  | Group | 0.520 | 0.637 | 0.051 | 0.678 |
|  |  |  |  |  |  |
|  | RB6 | 0.517 | <0.001 | 0.590 | <0.001 |
|  | RB7 | 0.622 | <0.001 | 0.609 | <0.001 |
| RG2 | RA5 | 0.518 | <0.001 | 0.611 | <0.001 |
|  | RA11 | 0.647 | <0.001 | 0.526 | <0.001 |
|  | Group | 0.581 | 0.682 | 0.585 | 0.921 |
|  |  |  |  |  |  |

The table contains correlation coefficients *r* with corresponding values of *p* for significance, for the relationship between the duty factor of burst of EMG activity of right muscle/muscle with SNC and the duty factor of burst of EMG activity of left muscle/control muscle obtained for the Sol and the EDL muscles in individual intact rats, saline and Riluzole treated animals. In addition the table contains the values of common correlation coefficients *r*_w_ with the values of *p*_w_ in the respective groups. Abbreviations: DF-duty factor, Sol-soleus, EDL-extensor digitorum longus.
