# Supplementary material for: Treatment with Riluzole Restores Normal Control of Soleus and Extensor Digitorum Longus Muscles during Locomotion in Adult Rats after Sciatic Nerve Crush at Birth
Source: PLoS One. 2017 Jan 17;12(1):e0170235. doi: 10.1371/journal.pone.0170235 (PMC5240973; doi:10.1371/journal.pone.0170235)
Supplement: S9 Table — The table contains mean (±SD) of interval durations, predicted durations, slopes and intercepts of regressions as well as correlation coefficients r with the values of p obtained in individual rats and in group of intact rats for the right muscles as well as in individual rats and in groups of saline and Riluzole treated animals for muscles with SNC. The values of SEM ranged from 1.19 to 3.83%. Abbreviations for statistical significance vs intact rats: *—p < 0.001. (DOC) [file pone.0170235.s009.doc]

**S9 Table. The duration of I_S-E_ and I_E-S_ intervals and the relationship with the burst duration of Sol muscle EMG activity.**

|  |  | I_S-E_ |  |  |  |  | I_E-S_ |  |  |  |  |
| --- | --- | --- | --- | --- | --- | --- | --- | --- | --- | --- | --- |
| Group | Rat | Duration | Slope | Inter-  cept | *r* | *p* | Duration | Slope | Inter-  cept | *r* | *p* |
|  |  | [ms] |  | [ms] |  |  | [ms] |  | [ms] |  |  |
|  |  |  |  |  |  |  |  |  |  |  |  |
|  | IN1 | 79±35 | 0.073 | 76 | 0.319 | 0.024 | 92±34 | 0.234 | 43 | 0.399 | 0.004 |
| IN | IN2 | 90±10 | 0.058 | 77 | 0.338 | 0.016 | 75±26 | 0.115 | 53 | 0.249 | 0.081 |
|  | IN3 | 89±11 | 0.063 | 74 | 0.298 | 0.035 | 74±25 | 0.128 | 54 | 0.263 | 0.064 |
| Group |  | 86±11 | 0.039 | 76 | 0.202 | 0.013 | 81±30 | 0.124 | 57 | 0.235 | 0.003 |
|  |  |  |  |  |  |  |  |  |  |  |  |
|  | NB4 | 105±24 | 0.091 | 85 | 0.302 | 0.033 | 199±94 | 0.942 | 1 | 0.900 | <0.001 |
|  | NB5 | 104±21 | 0.109 | 91 | 0.328 | 0.020 | 227±75 | 0.853 | 75 | 0.862 | <0.001 |
| 1S | NB2 | 71±25 | 0.060 | 60 | 0.362 | 0.009 | 168±63 | 0.680 | 36 | 0.906 | <0.001 |
|  | NB6 | 94±29 | 0.099 | 77 | 0.440 | 0.001 | 197±89 | 0.993 | 9 | 0.926 | <0.001 |
| Group |  | 93±24 | 0.096 | 76 | 0.322 | <0.001 | 198±85* | 0.753 | 45 | 0.746 | <0.001 |
| Predicted |  | 94±24 |  |  |  |  | 211±85* |  |  |  |  |
|  | NA4 | 99±12 | 0.069 | 77 | 0.342 | 0.015 | 178±47 | 0.977 | -10 | 0.949 | <0.001 |
|  | NA5 | 89±21 | 0.110 | 70 | 0.370 | 0.008 | 178±90 | 1.142 | -27 | 0.906 | <0.001 |
| 2S | NA7 | 81±14 | 0.026 | 77 | 0.110 | 0.446 | 203±63 | 0.907 | -1 | 0.851 | <0.001 |
|  | NA6 | 79±10 | 0.098 | 59 | 0.485 | <0.001 | 205±57 | 0.929 | -3 | 0.957 | <0.001 |
|  | KB6 | 126±19 | 0.059 | 83 | 0.210 | 0.143 | 273±71 | 0.917 | 42 | 0.858 | <0.001 |
| Group |  | 93±20 | 0.057 | 70 | 0.251 | <0.001 | 209±71* | 0.966 | -7 | 0.888 | <0.001 |
| Predicted |  | 92±20 |  |  |  |  | 191±71* |  |  |  |  |
|  | RA1 | 100±12 | 0.103 | 86 | 0.396 | 0.004 | 110±40 | 0.753 | 15 | 0.850 | <0.001 |
|  | RA4 | 91±10 | 0.088 | 76 | 0.471 | <0.001 | 133±45 | 0.762 | 10 | 0.918 | <0.001 |
| RG1 | RA6 | 87±16 | 0.065 | 83 | 0.277 | 0.051 | 134±46 | 0.643 | 33 | 0.854 | <0.001 |
|  | RB4 | 85±12 | 0.037 | 77 | 0.119 | 0.410 | 163±39 | 0.754 | 26 | 0.868 | <0.001 |
|  | RB5 | 88±14 | 0.075 | 73 | 0.417 | 0.002 | 195±81 | 0.963 | 8 | 0.931 | <0.001 |
| Group |  | 90±22 | 0.014 | 88 | 0.059 | 0.352 | 147±46* | 0.699 | 24 | 0.825 | <0.001 |
| Predicted |  | 89±22 |  |  |  |  | 167±46* |  |  |  |  |
|  | RB6 | 91±16 | 0.098 | 72 | 0.523 | <0.001 | 48±25 | 0.049 | 62 | 0.148 | 0.305 |
|  | RB7 | 94±24 | 0.080 | 82 | 0.318 | 0.024 | 84±27 | 0.053 | 64 | 0.158 | 0.273 |
| RG2 | RA5 | 96±19 | 0.156 | 74 | 0.597 | <0.001 | 35±10 | 0.025 | 21 | 0.170 | 0.237 |
|  | RA11 | 91±15 | 0.020 | 88 | 0.381 | 0.006 | 63±20 | 0.095 | 55 | 0.197 | 0.170 |
| Group |  | 93±16 | 0.108 | 79 | 0.449 | <0.001 | 59±20 | 0.047 | 45 | 0.188 | 0.007 |
| Predicted |  | 94±16 |  |  |  |  | 61±20 |  |  |  |  |
|  |  |  |  |  |  |  |  |  |  |  |  |

The table contains mean (±SD) of interval durations, predicted durations, slopes and intercepts of regressions as well as correlation coefficients *r* with the values of *p* obtained in individual rats and in group of intact rats for the right muscles as well as in individual rats and in groups of saline and Riluzole treated animals for muscles with SNC. The values of SEM ranged from 1.06 to 3.33%. Abbreviations for statistical significance vs intact rats: * - *p* < 0.001**.**
